# Genomic characterization and antifungal properties of *Paenibacillus polymyxa* YF, a promising biocontrol agent against *Fusarium oxysporum* pathogen of codonopsis root rot

**DOI:** 10.3389/fmicb.2025.1549944

**Published:** 2025-02-26

**Authors:** Ying Li, Xu Su, Wenjie Xi, Yanli Zheng, Yang Liu, Wangshan Zheng, Shiyu Wei, Yan Leng, Yongqiang Tian

**Affiliations:** ^1^School of Biological and Pharmaceutical Engineering, Lanzhou Jiaotong University, Lanzhou, China; ^2^Key Laboratory of Biodiversity Formation Mechanism and Comprehensive Utilization of the Qinghai-Tibet Plateau in Qinghai Province, Qinghai Normal University, Xining, China

**Keywords:** *Paenibacillus polymyxa*, *Codonopsis pilosula*, root rot, antifungal property, genomic characterization

## Abstract

Root rot, a destructive soil-borne disease, poses a significant threat to a wide range of economically important crops. Codonopsis, a high-value medicine plant, is particularly susceptible to substantial production losses caused by *Fusarium oxysporum*-induced root rot. In this study, we identified a promising biocontrol agent for codonopsis root rot, *Paenibacillus polymyxa* YF. *In vitro* assay demonstrated that the strain YF exhibited a 70.69% inhibition rate against *F. oxysporum* and broad-spectrum antifungal activities against the selected six postharvest pathogens. Additionally, the strain YF demonstrated significant plant growth-promoting properties. Subsequent *in vivo* inoculation assays revealed that the strain YF effectively mitigated disease symptoms of *F. oxysporum*-induced root rot in codonopsis, even achieving a complete disease prevention efficacy rate of 100%. Our findings further elucidated that the robust biocontrol capacity of the strain YF against *F. oxysporum* is mediated through multiple mechanisms, including inhibition of fusaric acid secretion, downregulation of virulence-associated genes in *F. oxysporum*, and the production of multiple hydrolytic enzymes. Genomic analysis showed that the strain YF has a 5.62-Mb single circular chromosome with 5,138 protein-coding genes. Comprehensive genome mining of the strain YF also identified numerous genes and gene clusters involved in bio-fertilization, resistance inducers synthesis, plant colonization, biofilm formation, and antimicrobial activity. These findings provide insights into the biocontrol mechanisms of the strain YF and offer substantial potential for its further exploration and application in crop production.

## 1 Introduction

Root rot, a highly destructive soil-borne disease, is caused by a diverse range of pathogens and poses a significant threat to global crop production (Nandris et al., 1987; Bodah, 2017). The symptoms of root rot initially develop underground, complicating early detection. Once symptoms appear above ground, plants typically do not recover, thus leading to serious crop losses worldwide (Rahman and Punja, 2005; Gossen et al., 2016). Fungal pathogens, particularly *Fusarium* spp., are the primary etiological agents of root rot, with *F. oxysporum* being a major contributor to the disease across various crops (Schneider et al., 2001; Okungbowa and Shittu, 2012; Gordon, 2017). The ability of these fungal pathogens to persist in plant debris and infested soils for extended periods, coupled with their increasing resistance to fungicides, makes them particularly challenging to control (Hamelin et al., 1996; Schneider et al., 2001; Grebenikova et al., 2018).

*Codonopsis pilosula* (Franch.) Nannf., commonly known as codonopsis, is cultivated widely for its therapeutic properties and tonic dual-effect (Dong et al., 2023). The dried root of codonopsis, called ‘Dang Shen’ in Chinese, has been used as a traditional medicine since the Qing Dynasty due to its medicinal properties (Gao et al., 2018). Contemporary pharmacological research has demonstrated that codonopsis exhibits a wide range of biological activities, including cardiovascular protection, immunity function regulation, hematopoiesis enhancement, mucosal protection and anti-ulcer cytotoxic and antibacterial effects, anti-tumor, anti-aging, and antioxidant capabilities (He et al., 2015; Dong et al., 2023). However, root rot disease severely damaged the quality and yield of codonopsis, with losses reaching up to 60% in some cases (Sun et al., 2020). *Fusarium* spp., particularly *F. oxysporum*, are the primary causal agents of codonopsis root rot, with an incidence rate of up to 70% in affected crops (Wang et al., 2011; Sun et al., 2020). *F. oxysporum* is also one of the most destructive pathogenic *Fusarium* species affecting many crops (Rabari et al., 2023). Current management strategies for *Fusarium* root rot rely heavily on chemical fungicides, but the emergence of fungicide resistance and the associated risks to human health and ecosystems have highlighted the urgent need for more sustainable and eco-friendly control measures.

Plant growth-promoting rhizobacteria (PGPR) are beneficial rhizospheric bacteria known for their ability to enhance plant growth and improve disease resistance (PGPR) (Lugtenberg and Kamilova, 2009; Beneduzi et al., 2012). The use of PGPR represents an environmentally sustainable approach to disease management and yield enhancement within the framework of sustainable agriculture (Bodah, 2017; Backer et al., 2018). PGPR promote plant growth through various mechanisms, including biological nitrogen fixation, phosphate solubilization, iron sequestration via siderophores, and modulation of phytohormone levels, which enhance seed emergence, increase plant biomass, and improve stress tolerance (Kang et al., 2019; Jiao et al., 2021; Dargiri et al., 2024). Additionally, PGPR protect plants against pathogens through direct mechanisms, such as the production of antimicrobial compounds, and indirect mechanisms, such as the induction of systemic resistance (ISR) in plants (Wang et al., 2024; Patel et al., 2020; Meena et al., 2020).

Due to their dual benefits, a diverse array of bacteria, including species of *Bacillus*, *Pseudomonas*, *Paenibacillus*, *Agrobacterium*, and *Trichoderma,* have been commercially utilized as effective biocontrol agents (BCAs) under both greenhouse and field conditions (Junaid et al., 2013; El-Saadony et al., 2022). Among these, many species of *Paenibacillus* are well-known plant-growth promoters and notable for enhancing the growth of many crops (e.g., rice, maize, tomato, ginseng, and Danshen), with various strains capable of promoting plant nutrient uptake, controlling phytopathogens, and producing phytohormones (Eastman et al., 2014; Grady et al., 2016). Moreover, as the type species of *Paenibacillus*, *P. polymyxa* were known as a reliable PGPR and stand out with its prominent biocontrol capabilities (Padda et al., 2017), which could enhance plant fitness through pathogen antagonism due to the production of ribosomally synthesized bacteriocins such as paeninodin which belong to lasso peptides that are naturally endowed with a broad spectrum of activity (Raza et al., 2008; Maksimov et al., 2012; Zhu et al., 2016), and paenilan that with antimicrobial activity against some Gram-positive bacteria (Zhu et al., 2016; Park et al., 2017). In addition, non-ribosomally synthesized lipopeptides (NRPs), such as polymyxin, fusaricidin, and tridecaptin, were first isolated from strains of *P. polymyxa*. Among these, polymyxins primarily disrupt the bacterial membrane causing lethality and are extensively used to treat infections of Gram-negative bacteria (Mohapatra et al., 2021), while the fusaricidins are active against fungi, including many important phytopathogens, and a variety of Gram-positive bacteria (Mülner et al., 2021), and tridecaptins show strong activity against Gram-negative bacteria (Cochrane and Vederas, 2016; Grady et al., 2016). Additionally, *P. polymyxa* enhance crop productivity by increasing nutrient availability and phytohormone production (Grady et al., 2016; Lal and Tabacchioni, 2009). Despite these advantages, there have been no reports on the use of Paenibacillus species for the biocontrol of codonopsis root rot.

Based on the above findings, this study aimed to identify an effective biocontrol strain for managing codonopsis root rot caused by *F. oxysporum* and to elucidate its inhibitory mechanisms. A *P. polymyxa* strain, designated YF, was isolated from sheep manure compost associated with healthy codonopsis plants and screened for its antifungal activity against *F. oxysporum*. Further investigations revealed the broad-spectrum antifungal activity of the strain YF against selected phytopathogenic fungi and the potential to promote plant growth of the strain YF. Greenhouse and field experiments demonstrated the efficacy of the strain YF in suppressing *F. oxysporum*-induced root rot and elucidated its underlying mechanisms. Genomic analysis of the strain YF identified numerous genes and gene clusters involved in the biosynthesis of antimicrobial peptides, ISR inducers, and factors relating to nutrient acquisition and phytohormone biosynthesis. These findings highlight the potential of the strain YF as a sustainable alternative to chemical fungicides for managing codonopsis root rot and suggest its broader applicability in agricultural and medical contexts.

## 2 Methods and materials

### 2.1 Isolation and identification of biocontrol strain

Preliminary field surveys indicated that the incidence of codonopsis root rot was lower in cultivation areas treated with natural sheep manure compost than those treated with chemical fertilizers alone, suggesting the presence of biocontrol-potent microorganisms in the sheep manure compost. Accordingly, the sheep manure compost samples were collected from the rhizosphere of healthy codonopsis in an herb plantation in Weiyuan County, Gansu Province, China (35°13′53.35″ N; 103°54′10.23″ E). The antagonistic strains were isolated by the dilution plating method. Subsamples (1 g) were serial dilutions up to 10^−5^ were proceeded by sterile distilled water, followed by incubation on a potato dextrose agar (PDA) plate at 35°C. Single colonies were purified by the streak plate method, and each of them was repeated three times. The *F. oxysporum* strain used in this study (GenBank ID: ITS, ON241791) was previously isolated and characterized from the roots of codonopsis plants exhibiting root rot symptoms by our laboratory, and its pathogenicity was confirmed. The antifungal activity of the candidate strain against *F. oxysporum* was evaluated using a confrontation assay. The pathogenic fungus was inoculated in the center of a PDA plate, and each candidate strain was inoculated at four equidistant points, 2.5 cm from the center. Only fungal pathogens served as a control. Colony diameters and inhibition zones were assessed after incubation at 28°C for 6 days. The inhibition percentage was derived from the comparison of mycelial diameters of *F. oxysporum* between control and treatment groups. Each experiment was conducted in triplicate. The morphology of the bacterial strain was examined using both optical microscopy and scanning electron microscopy (SEM). The accelerating voltage was 5 kV, and images were collected digitally from the emitted secondary electron signal. The 16S rRNA gene of the strain YF was amplified from genomic DNA using universal primers, and a maximum likelihood-based phylogenetic analysis inference was performed by Type (Strain) Genome Server (TYGS, https://tygs.dsmz.de) (Meier-Kolthoff and Göker, 2019).

### 2.2 Fermentation media optimization of the strain YF

The orthogonal matrix method was employed to optimize the appropriate components including the most suitable carbon, nitrogen, and mineral sources of fermentation media of the strain YF (Singh et al., 2017). Carbon sources tested included starch, glucose, maltose, sucrose, and yeast extract, while nitrogen sources included ammonium nitrate, ammonium chloride, ammonium sulfate, peptone, and dipotassium hydrogen phosphate. Mineral sources tested were calcium chloride, sodium chloride, magnesium sulfate, manganese chloride, and dipotassium hydrogen phosphate. Different media formulations were prepared based on LB medium and sterilized at 121°C and 2 bar pressure for 30 min. After cooling, the seed culture of the strain YF was inoculated into 250-mL flasks containing 100 mL of the corresponding media and incubated at 32°C on a rotary shaker at 180 rpm for 48 h. Optical density (OD) was measured at 600 nm to determine the CFU/mL of the strain YF. The one-factor orthogonal method was used to investigate the effects of medium components, and a Taguchi L_9_ (3^3^) orthogonal array was executed with three factors and three levels to determine the dosage of each component.

### 2.3 Assay of the broad-spectrum antifungal activity of the strain YF

In order to further test whether the strain YF had a broad-spectrum antifungal activity, we selected six phytopathogenic fungi that were preserved in our laboratory, including *F. acuminatum*, *F. equiseti*, *F. redolens*, *F. cotton*, *F. solan,* and *Colletotrichum gloeosporioides*. These phytopathogenic fungi were kept in our laboratory. The antifungal activity of the strain YF against each fungus was evaluated using the dual-culture assay described in section 2.1.

### 2.4 Colonization assays with the strain YF on seedling roots

The green fluorescent protein (GFP)-labeled *P. polymyxa* YF was constructed with a shuttle plasmid (pGFP4412), which introduced the plasmid-borne gfp and ampicillin-resistance genes into *P. polymyxa* strain YF.

The plasmid pGFP4412, purchased from Fenghui Biotechnology Co., Ltd. (China), was rejuvenated by inoculation into an LB medium. After activation, 100 mL of the strain YF culture was inoculated into a sterile LB liquid medium and incubated at 35°C with shaking at 180 rpm for 12 h. The bacterial culture was then centrifuged at 4,000 rpm for 2 min at room temperature to collect the cells. Plasmid DNA was extracted using a plasmid extraction kit and stored at −80°C.

To prepare competent cells of the strain YF, the activated strain was inoculated into 4 mL of GM I solution and shaken overnight at 32°C and 120 rpm for 12 h. The culture was then transferred to fresh GM I solution and incubated at 37°C for 4 h at 220 rpm. Subsequently, the culture was inoculated into 10 mL of GM II solution and incubated at 37°C at 220 rpm for 1.5 h. The bacterial cells were collected by centrifugation at 4,000 rpm for 5 min at room temperature, resuspended in 200 μL of GM II solution, and stored at −80°C. The heat shock method was used to transform the pGFP4412 plasmid into the strain YF. After heat shock at 45°C, 1 μL of pGFP4412 plasmid DNA was added to 200 μL of competent cells, followed by incubation at 37°C for 50 min and shaking at 180 rpm for 4 h. The transformed cells were plated on selective plates containing 100 μg/mL ampicillin, and single colonies were selected and verified using a laser confocal microscope after 2 days.

Codonopsis seedlings with root diameters of approximately 3 cm were cultivated in sterilized soil within flowerpots. Each pot was treated with 50 mL of a bacterial suspension at a concentration of 10^8^ CFU/mL and watered accordingly, while a control group received 50 mL of sterile water. Roots were harvested at 5, 10, 15, and 20 days post-inoculation and rinsed three times using PBS (1 M, pH 7.0). The roots were then sectioned into 1-mm slices for observation under a confocal laser scanning microscope (CLSM, Olympus FV3000, Olympus, Tokyo, Japan) with an excitation wavelength set at 488 nm. All bioassays and experimental procedures were replicated three times.

### 2.5 Growth-promoting effects of the strain YF

To quantify the production of indole-3-acetic acid (IAA) by the strain YF, the strain was cultured in LB broth at 30°C with shaking at 180 rpm. The culture supernatant was subjected to colorimetric IAA analysis using the Salkowski reagent. After centrifugation at 12,000 rpm for 5 min, the supernatant was mixed with Salkowski’s reagent in a 1:1 ratio, vortexed briefly, and incubated in the dark for at least 30 min at room temperature. The absorbance of the mixture was measured at 530 nm using a spectrophotometer. A standard curve was generated using serial dilutions of IAA (Sigma-Aldrich, USA) ranging from 0 to 20 mg/L. Each experiment was conducted in triplicate.

To evaluate the growth-promoting effects of the strain YF on codonopsis seedlings, sterile soil (~1 kg) was mixed with 20 mL of the strain YF suspension (1 × 10^8^ CFU/mL) and irrigated with 20 mL of the suspension every 2 weeks. The control group received sterile water only. After 60 days, plant height, root length, and root weight were measured. Each treatment consisted of 10 pots (one plant per pot).

### 2.6 Antagonistic effects of the strain YF against *F. oxysporum*

Codonopsis seedlings of uniform growth with a root diameter of ~0.5 cm were used to assess the antagonistic effects of the strain YF against *F. oxysporum*. Selected seedlings were sterilized in 75% (v/v) of alcohol for 15 s, subsequently immersed in 15% sodium hypochlorite solution for 1 min, and then rinsed three times with sterile water. Each root of codonopsis was slightly punctured in five places for subsequent usage. Negative and positive control groups were established, respectively. For the negative control, only inoculated with the mycelial plugs (5 mm diameter) taken from a 7-day-old *F. oxysporum* culture on the wound of codonopsis root. For the positive control group, carbendazim was sprayed onto the surface of the root. The treatment group was instead carbendazim by the suspension of the strain YF was sprayed onto the surface. All experimental procedures were conducted in triplicate. After incubation, codonopsis roots were placed in a dark moisturized growth chamber maintained at a temperature of 28°C for 4 days. The antagonistic potential of the strain YF was quantified by measuring the size of the lesion area of the codonopsis root. Root rot severity is classified based on the percentage of the diseased root system to the total root system (Winstead et al., 1952; Cong et al., 2018; Kolkman and Kelly, 2020): grade 0, no disease; grade 1, slight browning of the root system, with less than 15% discoloration; grade 3, partial browning, with 16–30% discoloration; grade 5, moderate browning, with 30–60% discoloration; and grade 7, severe browning, with more than 60% discoloration. The classification was calculated using the following formula:


$$\mathrm{Disease}\;\mathrm{rate}\%=\frac{\mathrm{Number}\;\mathrm{of}\;\mathrm{Disease}\;\mathrm{Plant}\;}{\mathrm{Total}\;\mathrm{Number}\;\mathrm{of}\;\mathrm{Assessed}\;\mathrm{Plant}\;}$$

$$\mathrm{Disease}\;\mathrm{incidence}\%=\frac{\sum \;\left(\mathrm{Disease}\;\mathrm{Grade}*\mathrm{Number}\;\mathrm{of}\;\mathrm{Disease}\;\mathrm{Plant}\;\right)\;}{\mathrm{Total}\;\mathrm{Number}\;\mathrm{of}\;\mathrm{Assessed}\;\mathrm{Plant}*\mathrm{Highest}\;\mathrm{Disease}\;\mathrm{Grade}\;}$$

According to the methods described above, we also assessed the therapeutic effects of the strain YF on *F. oxysporum* by calculating the disease incidence of the group which inoculated the mycelial plugs (5 mm diameter) of *F. oxysporum* on wounded codonopsis root firstly and then sprayed the suspension of the strain YF onto the surface after 48 h. Conversely, the preventive effects were assessed by calculating the disease incidence of the group that first sprayed the YF suspension onto the surface of wounded codonopsis roots, followed by inoculating the roots with the mycelial plugs (5 mm diameter) of *F. oxysporum* after 48 h. The control group was treated as above. All experimental procedures were conducted in triplicate.

To assess the disease control effects of the strain YF in the field, the roots of codonopsis were soaked in 50×, 100× and 150× diluted fermentation liquid of the strain YF before transplanting and irrigated by corresponding fermentation liquid every month until the mature stage of codonopsis. Moreover, carbendazim and sterile water were substituted for fermentation liquid as positive and blank control groups, respectively. The survival rate was measured in each experimental area (30 m^2^). However, the soil of the control group was only irrigated with sterile water. All experimental procedures were conducted in triplicate.

### 2.7 Biofilm formation assay

The capacity for biofilm formation by the strain YF was evaluated using crystal violet (CV) staining. First, 200 μL of bacterial suspension was inoculated onto a 96-well microtiter plate and cultured at 32°C for 60 h, and then, the biofilms attached to the bottom of the 96-plate were washed with distilled water five times and dried at 25°C overnight. The biofilms were then stained with 0.1% (w/v) crystal violet for 20 min, followed by the removal of excess stain with distilled water, while the bounded CV was dissolved with 200 μL of 33% acetic acid. Finally, the quantity of adherent bacteria was ascertained by measuring the optical density at 590 nm (OD_590_). The specific biofilm formation (SBF) (Naves et al., 2008) values were determined by using the formula: SBF = (B − NC)/BG, where B represents the OD_590_ of stained attached bacteria; NC, the OD_590_ of the stained control well (bacteria-free medium only), and BG, the OD_590_ of bacterial growth control. Each biofilm value was conducted in no fewer than three independent experiments. An SBF threshold of 0.5 was applied to categorize the isolates as biofilm formation positive (SBF value ≥0.5) or biofilm formation negative (SBF value <0.5).

### 2.8 Detection of antagonist-related lytic enzymes

The enzymatic activities of amylase, protease, cellulase, glucanase, chitinase, and pectinase of the strain YF were detected on agar plates containing starch, skim milk, sodium carboxymethyl cellulose, *β*-glucan, colloidal chitin, and poly-galacturonic acid, respectively. The antagonistic bacterial suspension (1 mL, OD_600_ = 1) was inoculated on five testing media in plates and cultured at 32°C for 3 days. The production of hydrolytic enzymes was inferred from the observation of bacterial colony growth and the dimensions of hydrolytic circles.

### 2.9 Effects of the strain YF on secretion of fusaric acid and expression of virulence genes of *F. oxysporum*

Ten mycelial plugs (5 mm diameter) of *F. oxysporum* were inoculated in 200 mL toxin-producing medium, followed by added 20 mL fermentation liquid of the strain YF, while the sterile culture medium was also added as blank control. After inoculation, the medium was maintained at 28°C for 12 days by shaking (160 r/min). Then, extracted with 200 mL ethyl acetate three times, and the organic solvents were collected and dissolved with ethyl acetate after vacuum drying at 40°C. The standard curve was created using fusaric acid solution with different concentrations (dissolved 0, 10, 20, 30, 40, and 50 μg fusaric acid into 1 mL ethyl acetate, respectively), and the wavelength of the maximum absorbance was detected by screening full wavelength. Moreover, the contents of our sample were calculated according to the standard curve.

Eight genes (*PL1*, *FPD1*, *SIX8*, *SIX1*, *Rho1*, *Fmk1*, *SNF1,* and *PelD*) that reportedly related to the virulence gene of *F. oxysporum* were selected to explore the effect of the strain YF on the whole dynamic process of *F. oxysporum* invading in the expression pattern of these genes (Covey et al., 2014). The selected genes were amplified using specific primers designed with the Primer5 software (Lalitha, 2000) (version 5.0), and the primer sequence of each gene as detailed in Supplementary Table S1. First, *F. oxysporum was* cultured in a PDA plate for 5 days for subsequent confront culture. The mycelial plugs (5 mm diameter) of *F. oxysporum* were inoculated on the left and right sides of the plate, followed by inoculation of the strain YF on the symmetric line of two plugs. Petri dishes were placed in an incubator at 25°C for 5 days, and the inoculation without the strain YF as a blank control. The total RNA of the hyphae of *F. oxysporum* was isolated with an RNA Mini-preps Kit according to the manufacturer’s protocol and was subsequently digested using the DNA enzyme. The cDNA was synthesized using the PrimeScript RT reagent kit, each experimental procedure was executed according to protocol. Then, the cDNA was used for the qRT-PCR using a 20 μL reaction mixture comprising 10 μL TB Green *Premix Ex Taq* II (Tli RNaseH Plus), 0.8 μL each of upstream and downstream primers (at a concentration of 0.4 μM), 2 μL of cDNA template, and 6.4 μL of ddH_2_O. Moreover, the *FEM1* gene was used as an internal reference.

### 2.10 Whole-genome sequencing and annotation

Genomic DNA was extracted from the strain YF cells with a QIAGEN Genomic DNA extraction kit according to the standard operating procedure provided by the manufacturer. High-quality DNA (OD260/280 = 1.8–2.0, >10 μg; NanoDrop2500, Thermo Fisher Scientific, Waltham, MA, United States) was used for sequencing. The whole genome sequencing and assembly were carried out by the Majorbio Bio-pharm Technology Co., Ltd., Shanghai, China. To obtain Illumina short reads, DNA libraries with 500-bp inserts were constructed and sequenced using an Illumina X-ten platform. In addition, high molecular weight DNA was prepared and used to construct PacBio SMRT Bell libraries, and the SMRT Bell libraries were sequenced using a PacBio Sequel system. Before estimating genome sizes, the short Illumina reads were filtered using fastp (v.0.20.0) (Chen et al., 2018) with default parameters. The clean reads were used to *de novo* assembly of the strain YF using Unicycler (Wick et al., 2017) (version 0.5.0), which generated a preliminary assembly based on short Illumina reads by using the algorithm of SPAdes, and then, the long PacBio reads were used to improve the quality of assembly by using the pipeline that combined the algorithms of miniasm and Racon. Finally, the polishing was performed with Pilonjin (version 1.22), and the completed bacteria genome mapping of the strain YF was generated. The circular map of the genome was generated utilizing Circos (Krzywinski et al., 2009) (version 0.64). Tandem Repeats Finder was employed to identify tandem repeats, and RepeatMasker (Chen, 2004) was deployed to detect repetitive elements through homology alignments with Repbase. Coding DNA sequence (CDS) prediction was performed using Glimmer (version 3.02). tRNAs and rRNAs were predicted using tRNAscan-SE (Lowe and Eddy, 1997) (version 2.0) and Barrnap (version 0.9), respectively. Functional annotation of protein-coding genes was performed by Barrnap (version 2.7.1+) (E value <1 × 10^−5^) using SwissProt (Boeckmann et al., 2003), NR, Pfam (Bateman et al., 2004), and the Gene Ontology (GO) database (Gene Ontology Consortium, 2004), respectively. Based on the string database, BLASTP (Boratyn et al., 2013) comparisons were also used to perform Clusters of Orthologous Groups of proteins (COG) and Kyoto Encyclopedia of Genes and Genomes (KEGG) annotation (Kanehisa et al., 2017).

### 2.11 Genome comparison and mining

Nineteen genome sequences of *Paenibacillus* spp. were procured from GenBank. The accession numbers are detailed in Supplementary Table S2. The average nucleotide identity (ANI) and digital DNA–DNA hybridization (DDH) values were computed by using JSpeciesWS (Richter et al., 2016) and Genome-to-Genome Distance Calculator (Meier-Kolthoff et al., 2022) (GGDC, version 3.0) online services, respectively. The heatmap of the ANI and DDH matrix was generated with TBtools (Chen et al., 2020) (version 1.6). A phylogenetic analysis based on multiple conserved gene sequences of *Paenibacillus* spp. by using the Automated Multi-Locus Sequence Analysis (autoMLSA) web server.^1^ The syntenic analysis between pairs of genomes was searched for by the MAUVE (Darling et al., 2004). Attributes contributing to plant growth were analyzed by employing the PLaBAse (Patz et al., 2021) web server. Biosynthetic gene clusters for secondary metabolites (containing antibiotics) in *P. polymyxa* YF were identified through antiSMASH (Medema et al., 2011) (version 7.1.0) online services. Additional genes implicated in plant growth promotion and antibiotic production were screened according to precedent literature. BLAST (Boratyn et al., 2013) was employed to assess the homology of the genes or gene clusters between YF and other strains.

## 3 Results

### 3.1 Isolation and screening of antagonistic strain YF

**Figure 1:**
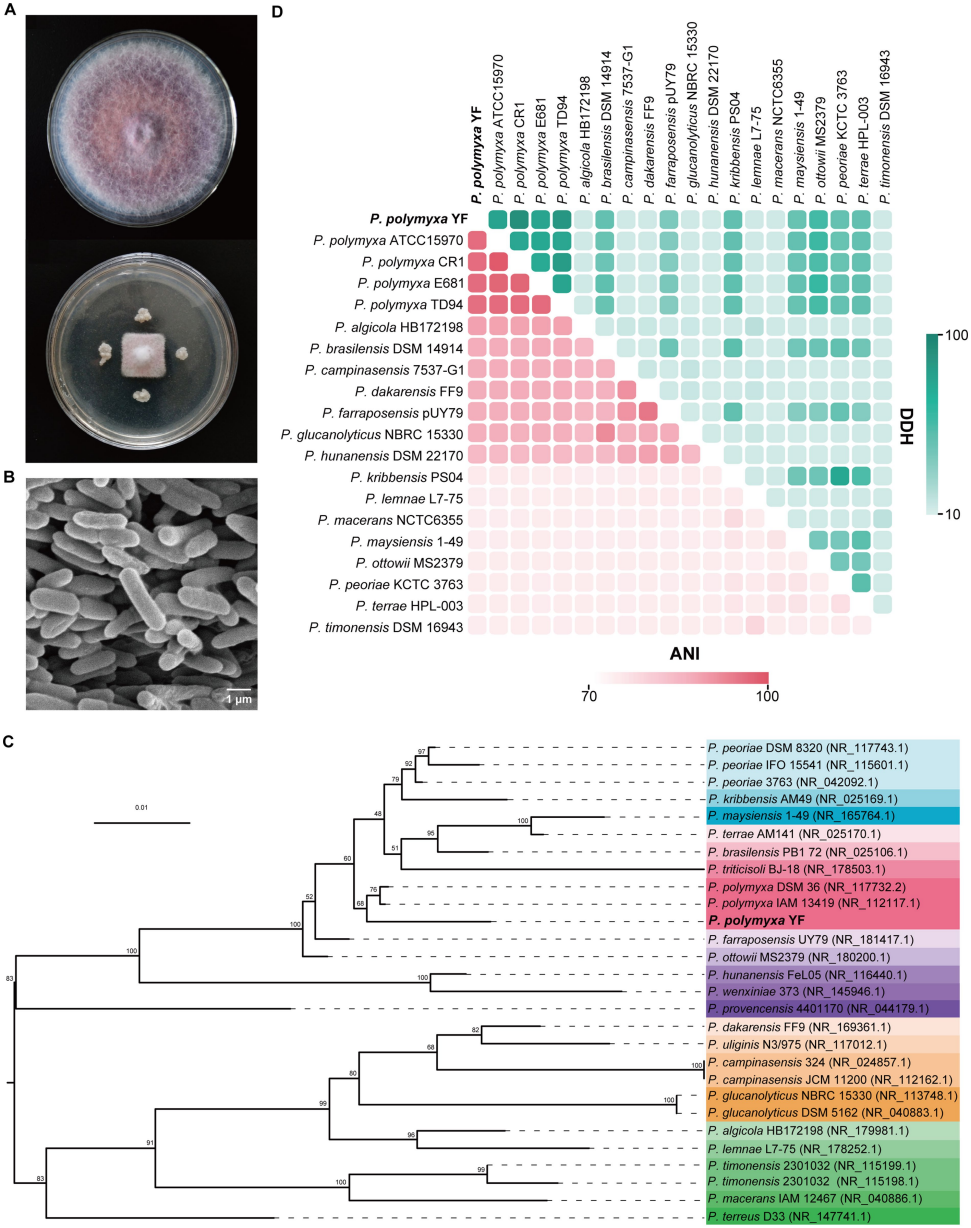
Antagonistic activity and identification of the strain YF. **(A)** Growth inhibition of *F. oxysporum* after being treated with the strain YF. Plates inoculated only with *F. oxysporum* were used as a control. **(B)** Morphological characteristics of the strain YF. **(C)** The maximum-likelihood phylogenetic tree of the strain YF based on 16S rRNA gene sequences. Numbers listed at the branches were bootstrap values based on 1,000 replications. Bar: 0.01 substitutions per nucleotide position. **(D)** Heat map of average nucleotide identity (ANI) values and DNA–DNA hybridization (DDH) values compared among YF and 19 related strains. ANI and DDH values are indicated by the color intensity.

The strain YF was isolated from sheep manure compost of healthy codonopsis, and it exhibited excellent antibacterial activities against *F. oxysporum* with a pronounced inhibition ratio of 70.69% (Figure 1A). Colonies of the strain YF on the PDA plate are milky white, circular, raised, tacky and opaque, with a wet and wrinkled surface after a 48-h incubation period at 35°C (Supplementary Figure S1A). Optical microscopic examination revealed that the cells were Gram-positive (Supplementary Figure S1B), spore-forming, and ovoid-rod-shaped, as confirmed by SEM analysis (Figure 1B). The optimal liquid culture for the strain YF consisted of 15 g sucrose, 15 g peptone, 5 g magnesium sulfate, 3 g sodium chloride, and 1 L water (Supplementary Table S3).

The 16S rRNA gene of the strain YF was amplified firstly by using universal primers, and the sequence exhibited high homology with the 16S rRNA gene of multiple *Paenibacillus* spp. in GenBank databases. Of which, *P. polymyxa* strain DSM36 showed the highest homology with the strain YF at 98.87%, followed by 98.80 and 98.59% similarity to *P. peoriae* strain 3,763 and *P. polymyxa* strain IAM13419, respectively (Supplemetnary Table S4). The phylogenetic analysis was also conducted using the maximum-likelihood method based on 16S rRNA genes, the result demonstrated that the isolated strain YF appeared to belong to *P. polymyxa* and formed a well-delineated subclade with strains *P. polymyxa* DSM 36, and *P. polymyxa* IAM 13419 (Figure 1C). Moreover, a phylogenetic tree constructed based on a housekeeping gene *gyrB* showed that *P. peoriae* nested into the cluster of *P. polymyxa*, and the strain YF was sister to *P. polymyxa* F1 (Supplementary Figure S2A). Additionally, 81 conserved genes were identified through autoMLST analysis, and phylogenetic tree construction using concatenated gene matrices further supported that the strain YF is classified as *P. polymyxa* (Supplementary Figure S2B).

Both ANI and DDH are recognized as robust methods for assessing evolutionary distance assessment between bacterial species. Thus, ANI and DDH values were calculated between the genome sequence of the strain YF and multiple *Paenibacillus* sp. (Figure 1C and Supplementary Table S5). All ANI values between the strain YF and multiple strains of *P. polymyxa* are in the range of 95.96 to 96.37% (Figure 1D and Supplementary Table S5), surpassing the species demarcation threshold of 95% for species demarcation. Similarly, the DDH values between the strain YF and multiple *Paenibacillus* sp. are in the range of 74.9–78.3% (Figure 1D and Supplementary Table S5). These values were also more than the accepted species threshold of 70%. Synthetically considering the phenotypic, physiological, biochemical, and phylogenetic characteristics, the strain YF was referred to as *P. polymyxa*.

### 3.2 Broad-spectrum antifungal activity of the strain YF

**Figure 2:**
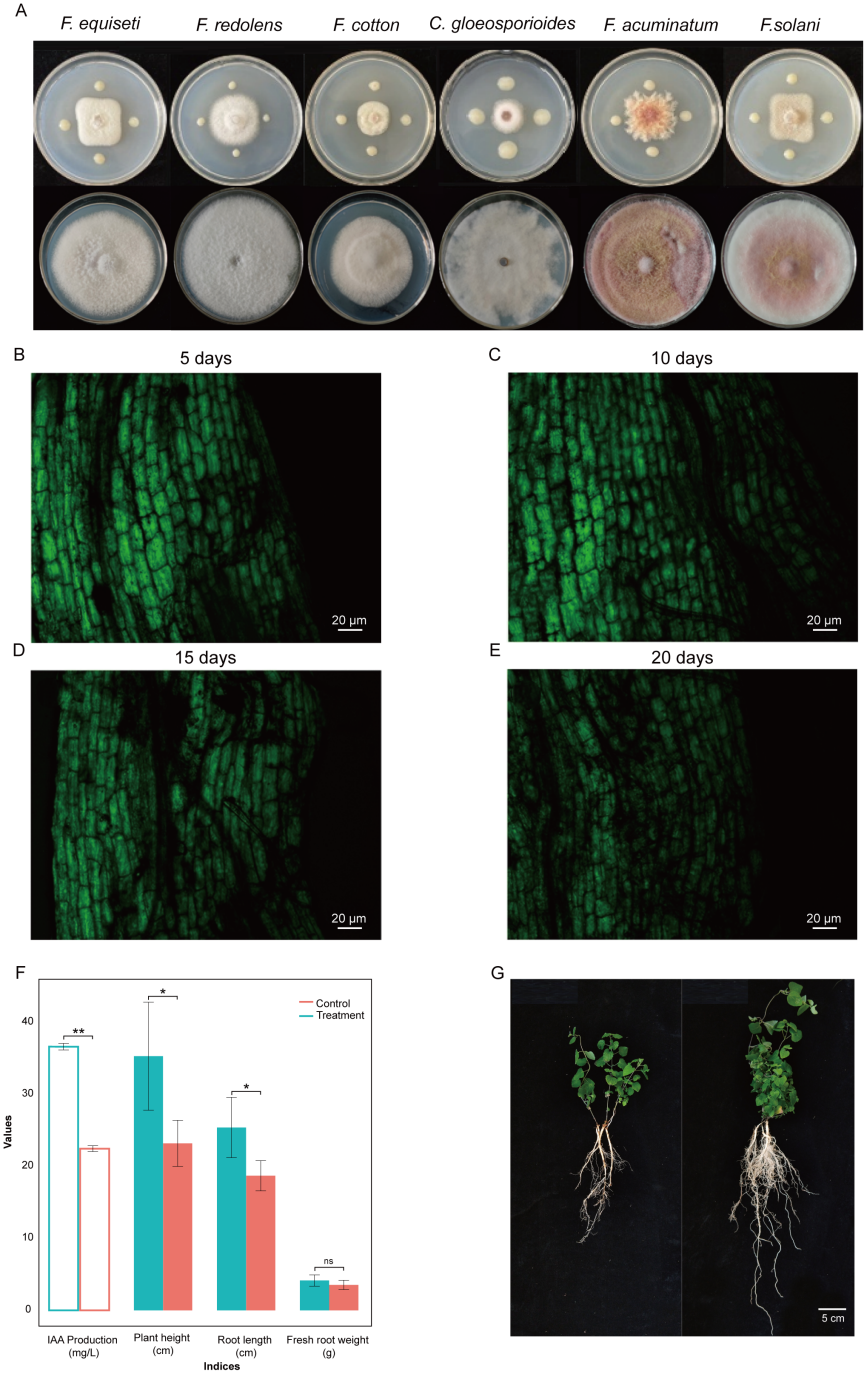
Broad-spectrum antagonistic activity **(A)** and colonization of the strain YF-pGFP in the roots of codonopsis for 5 days **(B)**, 10 days **(C)**, 15 days **(D)**, and 20 days **(E)**, respectively. GFP fluorescence exhibits excitation at 485 nm. The ability of IAA production and the effect on plant growth parameters of YF compared to controls **(F)**. An illustration of plant growth promoting eficiency on codonopsis seedlings **(G)**, left is the control group that right is the treatment group that irrigated with suspension of strain YF.

In addition to its activity against *F. oxysporum*, the primary causal agent of Codonopsis root rot, the strain YF demonstrated broad-spectrum antifungal activity against six phytopathogenic fungi: *F. acuminatum*, *F. equiseti*, *F. redolens*, *F. cotton*, *F. solan,* and *C. gloeosporioides* (Figure 2A). The inhibition rates ranged from 57.14 to 69.12%, and the strongest inhibition rate was detected against *C. gloeosporioides*, while the lowest inhibition rate was observed against *F. redolens*. These results indicated that *P. polymyxa* YF can be used as a broad-spectrum antifungal biocontrol agent and is valuable for further study.

### 3.3 Biofilm formation and colonization of the strain YF

Biofilm formation and subsequent colonization on the root area are crucial steps and preconditions for exerting beneficial effects on plant growth; thus, the processes of colonization were also explored in the strain YF. The ability to form a biofilm of the strain YF was measured by using the SBF index based on the CV staining. The results showed that the submerged biofilm formed after 72 h of cultivation in the medium, the absorbance value was 0.82 and 0.117 at 590 nm for adherent and stained bacteria samples and the control group (containing only bacteria-free medium), respectively, and the corresponding OD_600_ values for the suspension of the strain YF was 0.5. Calculated from the formula SBF = (B-NC)/BG, the SBF index of the strain YF was 1.406. These results substantiate the capacity of the strain YF in biofilm formation.

The colonization pattern of the strain YF was visualized and tracked by using green fluorescent protein (GFP)-labeled YF strains. Plasmid pGFP4412 was introduced into the YF strain via heat shock, and the transformants were preliminarily screened on selective plates containing 80 μg/mL Ampicillin. Randomly selected colonies from selective plates were used to detect the expression of GFP in the strain YF by CLSM. The observation showed that the GFP-labeled YF emits bright green fluorescence. Moreover, the *in vitro* growth patterns of the GFP-tagged YF and the wild-type YF strain were assessed over a 24-h period. Compared to wild-type growth, the transformation of pGFP4412 did not markedly influence the growth dynamics of bacteria, as depicted in Supplementary Figure S3.

After confirming the transformation of the GFP-labeled YF strains, we observed the dynamic of GFP-labeled YF strains during colonizing surfaces of codonopsis roots at different times (5, 10, 15, and 20 days after inoculation) with their cultures in the soil (Figures 2B–E and Supplementary Figures S3–S5). Five days after inoculation, it was found that GFP-labeled YF strains already colonized on the surface of codonopsis roots (Figure 2B). Ten days after inoculation, the colonies with the brightest fluorescence indicated the highest colonization density of GFP-labeled YF strains (Figure 2C). However, as the days of colonization increase, fluorescence showed a decreasing trend at 15 and 20 days after inoculation, and the density colonization of GFP-labeled YF strains was decreased obviously at 20 days after inoculation (Figures 2D,E). Our results further confirmed that GFP-labeled YF strains could stably colonize on codonopsis roots, and the maximum population number was reached approximately 10 days after inoculation (Figures 2B–E).

### 3.4 Growth-promoting effect of the strain YF

As illustrated in Figure 2, the IAA production by the strain YF in Trp-containing and Trp-free media was quantified as 28.39 mg/L and 16.22 mg/L, respectively. These results indicated that the strain YF with the ability to produce IAA by tryptophan (Figure 2F).

As the strain with the ability of IAA production, we further investigated the effect on plant growth. Greenhouse experiments revealed that the strain YF significantly enhanced codonopsis growth parameters compared to controls (Figures 2F, G). Treated plants that irrigated with suspension of the strain YF showed increased height (25.4 ± 4.2 cm vs. 17.8 ± 1.5 cm), root length (35.3 ± 7.5 cm vs. 22.2 ± 1.8 cm), and fresh root weight (4.15 ± 0.78 g vs. 3.54 ± 0.65 g). Furthermore, the plant height and root length were significantly higher in the treatment group than in the control group. Both of the above results collectively suggested that the strain YF has a positive influence on the growth of codonopsis.

### 3.5 Disease control effects of the strain YF against *F. oxysporum*

As shown in Figure 1A, the strain YF exhibited antagonistic activity against *F. oxysporum in vitro*. To further verify the potential of the strain YF to suppress root rot on *F. oxysporum*, we conducted greenhouse and field experiments to evaluate the antagonistic effects of the strain YF treatment on codonopsis root rot.

**Figure 3:**
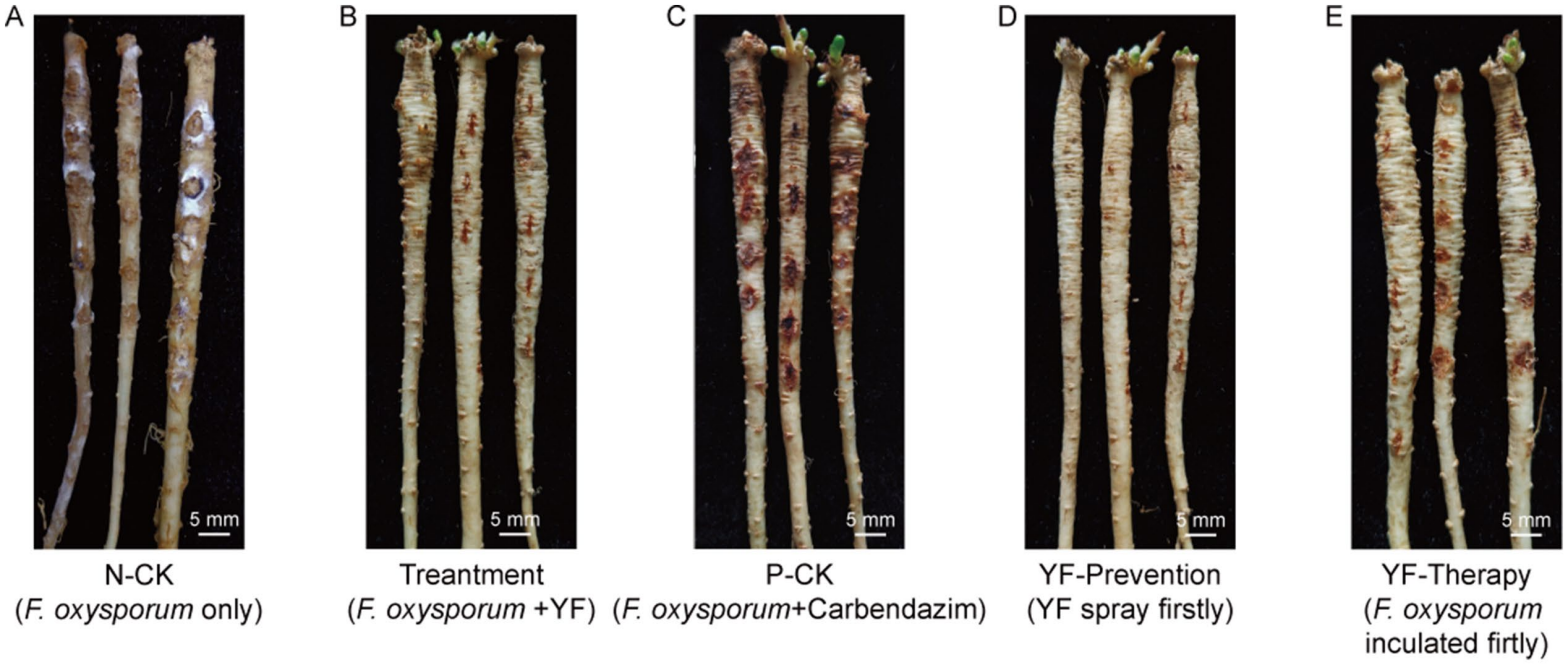
An illustration of disease control efficiency on root rot of codonopsis. **(A)** The negative control group *(N-CK)* that only inoculated with *F. oxysporum*. **(B)** The treatment group that inoculated with *F. oxysporum* and then sprayed the suspension of YF onto the surface. **(C)** The positive control group *(P-CK)* inoculated with *F. oxysporum*, and then treated with chemical fungicide (*F. oxysporum* + carbendazim). **(D)** The prevention group that sprayed the suspension of YF 48 h before inoculated with *F. oxysporum* (YF + *F. oxysporum*). **(E)** The therapy group that sprayed the suspension of YF 48 h after inoculated with *F. oxysporum* (*F. oxysporum* + YF).

The disease index of the negative control group (N-CK) that codonopsis plant roots were only inoculated with *F. oxysporum* was 58.66 ± 2.5 (Figure 3A and Table 1), while the treatment group, where the codonopsis root was inoculated with *F. oxysporum* and then sprayed the suspension of the strain YF onto the surface (*F. oxysporum* + YF), registered a disease index of 6.33 ± 1.7 (Figure 3B and Table 1), indicated an 89.2% disease control effects of the strain YF. In the same way, the positive control group (P-CK) (Figure 3C and Table 1), where codonopsis roots were inoculated with *F. oxysporum* and treated with chemical fungicide (*F. oxysporum* + carbendazim), exhibited a disease index of 19.67 ± 2.7 and a control effect of 66.5%. Figures 3A–C graphically contrast the disease control effects of the strain YF when compared with the positive and negative control groups. The results showed that codonopsis plants in the negative control groups, inoculated with *F. oxysporum* displayed extensive root rot lesions, whereas those treated with *F. oxysporum* + YF only with slight incidence of root rot disease. Similarly, there was weaker root rot disease in the positive control group (P-CK) when a chemical fungicide, was used after *F. oxysporum* inoculation.

**Table 1:** Statistics on the inhibition rate of YF against *Fusarium oxysporum.*

Treatment	Disease index	Control effect (%)
N-CK (only *F. oxysporum*)	58.66 ± 2.5a	–
Treatment (*F. oxysporum* + YF)	6.33 ± 1.7c	89.2
P-CK (*F. oxysporum* + carbendazim)	19.67 ± 2.7b	66.5
Prevention (Inoculate YF before *F. oxysporum*)	0c	100
Therapy (Inoculate YF after *F. oxysporum*)	9.95 ± 2.1b	83.0

In addition, we evaluated the preventive effects and therapeutic effect of the strain YF treatment on root rot of codonopsis as follows: the codonopsis plant roots were sprayed with the suspension of the strain YF 48 h before being inoculated with *F. oxysporum* (YF-Prevention) for evaluation of preventive effect, and codonopsis roots were sprayed with the suspension of the strain YF 48 h after being inoculated with *F. oxysporum* (YF-Therapy) for the evaluation of therapeutic effect, also, *F. oxysporum* only were used as control (same as N-CK above). Surprisingly, the disease index of the prevention group was 0, indicating 100% effectiveness in disease prevention and control of the strain YF (Figure 3D and Table 1). Moreover, the YF-The treatment significantly reduced the severity of root rot with a disease index of 9.95 ± 2.1 and 83% therapeutic effect (Figure 3E and Table 1). The illustrative representation showed significantly reduced lesion areas on the surface of codonopsis which were treated with the suspension of the strain YF in comparison with that of the control. Therefore, the strain YF was effective in managing the root rot disease of codonopsis.

A field trial was also executed to investigate the biocontrol efficiency strain YF in root rot caused by *F. oxysporum*. Three doses of the strain YF fermentation liquid were applied to measure the inhibitory activity against *F. oxysporum*, the treatment group involved soaking the roots of codonopsis in different dilutions of the strain YF fermentation liquid before planting, and the codonopsis plants were irrigated with corresponding dilutions of the fermentation liquid in the soil every month until harvest. The survival rate is then calculated and compared with the positive control group (treated with chemical fungicide) and the blank control group (Supplemetnary Table S6). The survival rate of codonopsis plants was 35.10 and 41.72% in the blank control and positive control group, respectively, while in the three treatment groups of 50×, 100×, and 150× diluted fermentation liquid, 44.24, 52.07, and 51.31% of survival rate were detected, respectively (Supplemetnary Table S6), indicated the most powerful biocontrol efficiency of 100× diluted fermentation liquid of YF for codonopsis root rot disease.

Collectively, these findings demonstrate that the root rot symptoms of codonopsis plants in both the greenhouse and the field that induced by *F. oxysporum* were effectively control by strain YF.

**Figure 4:**
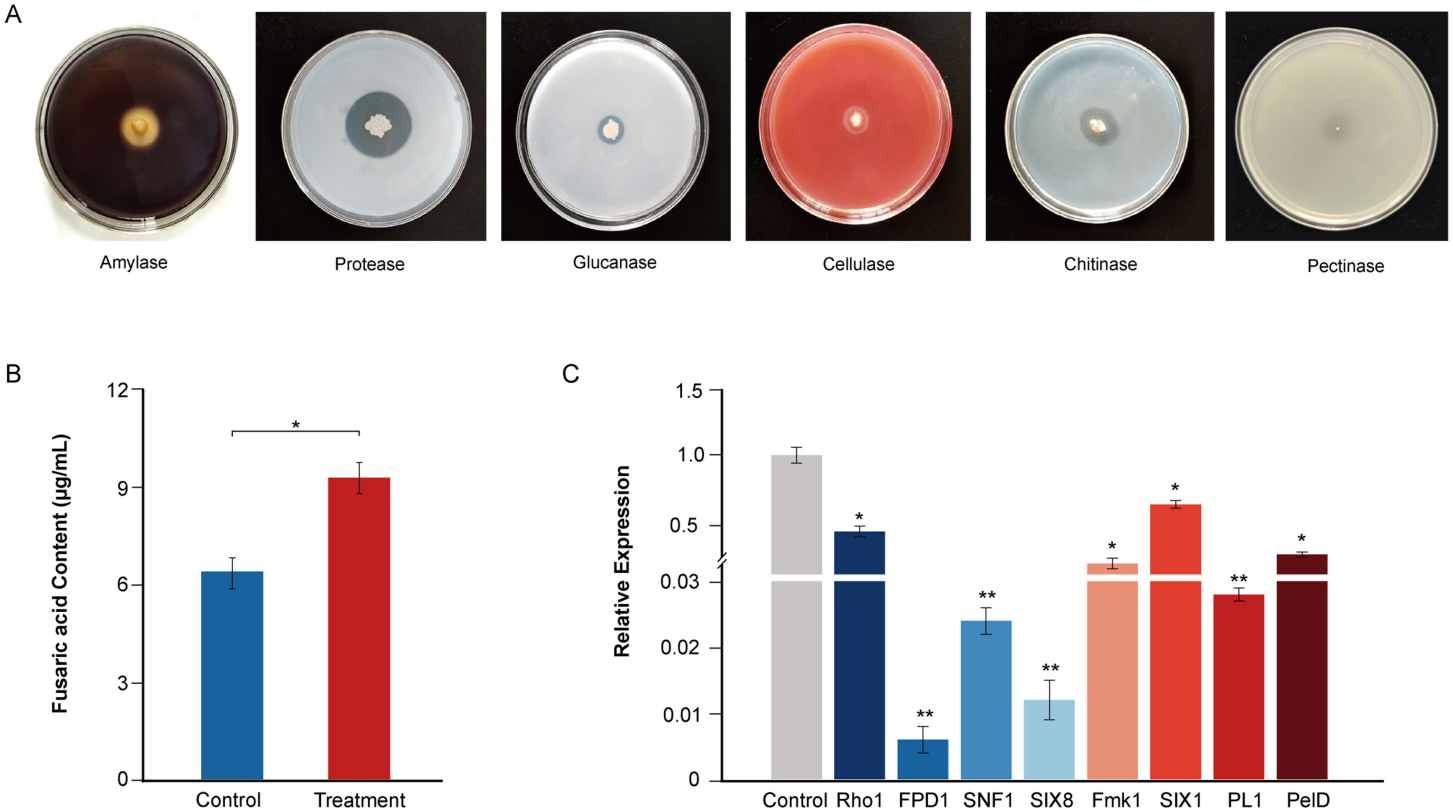
Plate clearance assay for extracellular enzyme activity and antifungal mechanisms of the strain YF. **(A)** Extracellular activity demonstrated by the strain YF. Zone of clearance indicates hydrolysis of the corresponding substrate, in order from left to right are amylase, protease, glucanase, cellulase, and chitinase. **(B)** Effect of the strain YF on secretion of fusaric acid in *F. oxysporum*. **(C)** Detection of virulence-related gene expression levels in *F. oxysporum* treated with the strain YF. The values in Figures 5B,C represent the means ± standard deviation (SD) of three independent samples.

### 3.6 Antifungal mechanisms of the strain YF

To further reveal the antagonistic mechanism of the strain YF, the type of extracellular lytic enzyme that contributes to the antifungal activities of the strain YF was screened by a selective medium (Figure 4A). The results showed that translucent hydrolysis circles were formed around colonies of the strain YF on the mediums indicating that the strain YF could produce multiple antagonist related lytic enzymes, including amylase, protease, cellulase, glucanase, and chitinase (Figure 4A), which further enhance its ability to break cell wall and leading to cell lysis of pathogens. However, there are nearly no translucent hydrolysis circles around colonies of the strain YF on the medium with PGA as the only carbon source, indicating that the strain YF could as a safe BCA for plant disease management (Figure 4A).

**Figure 5:**
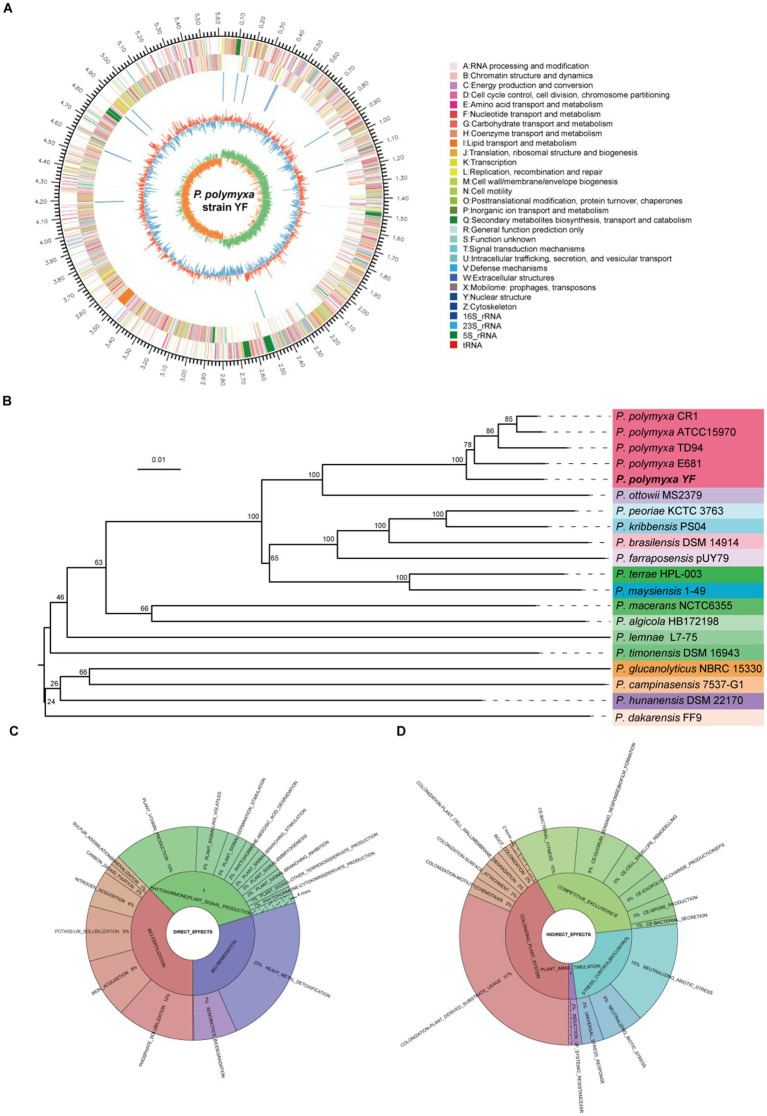
Genome analyses of the strain YF. **(A)** Genome map of the strain YF, the outermost circle of the diagram represents the genome size; the second and third circles indicate the CDS on the forward and reverse strands, respectively, with different colors denoting the functional classifications of CDS according to the COG categories; the fourth circle shows the locations of rRNA and tRNA; the fifth circle represents the GC content, where the red portions extending outward indicate regions with a GC content higher than the average GC content of the entire genome. Conversely, the blue portions extending inward represent regions with a GC content lower than the average. The innermost circle displays the GC skew values. **(B)** phylogeny and genome features—TYGS genome-based phylogeny tree; Krona plot of direct **(C)** and indirect **(D)** plant growth promoting traits observed in the strain YF from PLaBAse web server.

Moreover, to gain further insights into the antifungal mechanism of the strain YF, we conducted a confrontational culture between the strain YF and *F. oxysporum*. We analyzed the content of its toxin fusaric acid and the expression patterns of its pathogenic genes. The results showed that significantly inhibited fusaric acid production when compared with the control group. After treatment of the strain YF fermentation liquid, the production of fusaric acid was reduced to 6.35 μg/mL and significantly lower than that in the control group was 9.21 μg/mL, indicating the strain YF possesses the ability to inhibit secretion of fusaric acid (Figure 4B). Expression pattern of the key genes involved in colonization and hyphal development during *F. oxysporum* invasion were also detected after confrontational culture with the strain YF using qRT-PCR. In support, we detected significantly downregulated expression in the *F. oxysporum* of key genes involved in infection (*SNF1*, *PL1*, *Fmk1,* and *Rho1*), colonization (*SIX8* and *SIX1*), pathogenicity (*PelD*), and hyphal development (*FPD1*) (Figure 4C and Supplementary Table S7). We used the FEM1 gene as a reference gene to analyze the virulence gene quantitatively, and the fold change in gene expression of *FPD1*, *SIX8*, *SNF1*, *PL1*, *Fmk1*, *PelD*, *Rho1,* and *SIX1* are 0.006, 0.012, 0.024, 0.028, 0.182, 0.250, 0.423, and 0.629, respectively. These results provided insight into the mechanisms of the strain YF to inhibit the pathogenicity of *F. oxysporum*.

### 3.7 Genome features and comparative genomics analysis

Both the next-generation sequencing and the third-generation sequencing were adopted to produce a complete genome sequence of the strain YF. In total, 1.17 Gb of raw Illumina reads were generated, and after quality control, 1.16 Gb (~197× depth) of high-quality clean reads with a mean Q30 of 94.42% were obtained (Supplemetnary Table S8). In addition, 0.30 Gb PacBio reads (~51× depth) were generated in 315,34 reads with a mean and N50 length of 10,055 bp (Supplemetnary Table S9). The complete genome sequence of *P. polymyxa* YF was produced by combining the highly accurate and long read lengths of technologies and comprised a circular 5,615,829-bp chromosome, with an average GC content of 45.74% (Figure 5A). In total, 5,301 genes were annotated, including 5,138 protein-coding genes (Supplemetnary Table S10), 107 tRNA genes, and 56 sRNA genes. In addition, a total of 39 ribosomal RNA operons were present on the chromosome: 13 5S rRNAs, 13 16S rRNAs, and 13 23S rRNAs. Ten putative gene islands (GIs) were found in the strain YF using the GI prediction methods, and the size of GIs ranged from 12.5 to 80.4 kb (Supplemetnary Table S11). Clustered Regularly Interspersed Short Palindromic Repeats (CRISPRs) contain multiple short and repeated sequences, eight CRISPRs were involved in the strain YF, and the length of repeated sequences ranged from 162 to 2,645 bp (Supplemetnary Table S12). The phylogenetic analysis showed that the strain YF was clearly clustered with other strains of *P. polymyxa* and located in the basal of the clade (Figure 5B). Moreover, both phylogenetic analysis and genome-to-genome alignment (Supplementary Figure S6) showed that among the different strains *of P. polymyxa*, the strain YF showed the highest similarity with E681. The functional annotation of the whole genome of the strain YF was performed utilizing six different gene annotation databases (Supplementary Figure S7). There was a total of 5,091 annotations discovered in the NR database, which represents 99.09% of the total number of annotations. In addition, the Pfam, COG (Supplementary Figure S8), Swiss-Prot databases, in that order, had 4,259 (82.89%), 4,134 (80.46%), and 3,839 (74.72%), whereas the GO (Supplementary Figure S9) and KEGG (Supplementary Figure S10) databases contained 2,979 (57.98%) and 2,820 (54.86%) annotations, respectively. Of which, 23 groups were created from COG database, and 41 categories were created from KEGG database. The CAZy database contained a total of 270 annotations, which included glycoside hydrolases (138), glycosyltransferases (60), carbohydrate esterases (45), auxiliary activities (7), carbohydrate-binding modules (8), and polysaccharide lyases (12) (Supplementary Figure S11).

### 3.8 Genome mining for genes and gene clusters for plant growth promotion and antibiotic synthesis

Beneficial bacteria promote plant growth by facilitating nutrient uptake and phytohormone production, with numerous genes associated with plant growth promotion and protection identified in the strain YF (Table 2). The strain YF genome harbors seven genes implicated in indole-3-acetic acid (IAA) biosynthesis, including those encoding the putative indole pyruvate decarboxylase (*IpdC*), a pivotal enzyme in the IPyA pathway, indicating the capability of the strain YF to produce IAA. Nitrogen and phosphorus are essential for plant growth and development, yet bioavailability of them in the soil is a major limiting factor for plant growth. One molybdenum (Mo)-dependent nitrogenase (Nif) gene cluster, comprising nine genes (*nifB*, *nifH*, *nifD*, *nifK*, *nifE*, *nifN*, *nifX*, *hesA*, and *nifV*) was predicted in the strain YF and is hypothesized to participate in nitrate assimilation and reduction. Additionally, a gene cluster implicated in phosphate solubilization, consisting of *pstS*, *pstC*, *pstA,* and *pstB*, was identified in the strain YF genome. In addition, five genes (including two *mgtE* genes and three *corA* genes) and three gene members of the *kpd* gene family were predicted to be involved in magnesium utilization and potassium transporter, respectively.

**Table 2:** Information of the genes related to plant growth promotion in the YF genome.

Gene name	Location		Function
*IpdC*	1,634,953	1,636,698	IAA synthesis
*trpA*	3,186,992	3,186,186	
*trpB*	3,188,185	3,186,989	
*trpC*	3,189,644	3,188,850	
*trpD*	3,190,680	3,189,634	
*trpE*	3,192,265	3,190,715	
*nifH*	1,201,882	1,202,748	Nitrate transport and reduction
*nifD*	1,202,837	1,204,285	
*nifK*	1,204,282	1,205,811	
*nifE*	1,205,892	1,207,253	
*nifN*	1,207,243	1,208,550	
*nifX*	1,208,547	1,208,936	
*hesA*	1,209,040	1,209,804	
*nifV*	1,209,782	1,210,918	
*pstS*	1,836,132	1,837,055	Phosphate solubilization
*pstC*	1,837,162	1,838,094	
*pstA*	1,838,094	1,838,990	
*pstB*	1,839,008	1,839,850	
*mgtC*	1,254,983	1,255,654	Magnesium utilization
	4,569,502	4,568,789	
*corA*	969,790	970,749	
	1,673,544	1,672,609	
	4,586,079	4,585,120	
*kdpA*	1,474,143	1,475,819	Potassium transporter
*kdpB*	1,475,851	1,477,887	
*kdpC*	1,477,907	1,478,635	

Furthermore, the PLaBAse web server analysis of the strain YF showed the presence of various direct (Figure 5C) and indirect effects (Figure 5D) associated with plant growth promotion. Direct effects encompassed genes related to bio-fertilization (Supplementary Figure S12), phytohormones (Supplementary Figure S13), and bioremediation (Supplementary Figure S14). Indirect effects comprised genes associated with the plant colonization system (Supplementary Figure S15), competitive exclusion (Supplementary Figure S16), and stress control (Supplementary Figure S17). *Paenibacillus* species with numerous biocontrol capabilities including elicit-induced systemic resistance (ISR) and pathogen or microbe-triggered immunity (PTI/MTI) (Lal and Tabacchioni, 2009; Grady et al., 2016). As detailed in Table 3, genes encoding several elicitors, such as 2, 3-butanediol, acetoin, peptidoglycan, and EF-Tu, were detected in the strain YF, indicating its potential to induce resistance in plants.

**Table 3:** Information of the genes involved in the synthesis of resistance inducers in the strain YF genome.

Gene name	Location	Gene product	Plant resistance type
*alsS*	2,333,518	2,331,824	Acetolactate synthase	Induced systemic resistance
*alsD*	2,331,661	2,330,915	Acetolactate decarboxylase	
*ispE*	35,091	35,945	4-diphosphocytidyl-2-C-methyl-D-erythritol kinase	
*ispF*	4,894,566	4,894,090	2-C-methyl-D-erythritol 2,4-cyclodiphosphate synthase	
*ispD*	4,895,261	4,894,563	2-C-methyl-D-erythritol 4-phosphate	
*dacA*	4,832,925	4,832,092	Carboxypeptidase	Pathogen or microbe-triggered immunity
*flgL*	5,030,227	5,029,307	Flagellin	
*tuf*	4,871,459	4,870,269	Elongation factor Tu	

**Figure 6:**
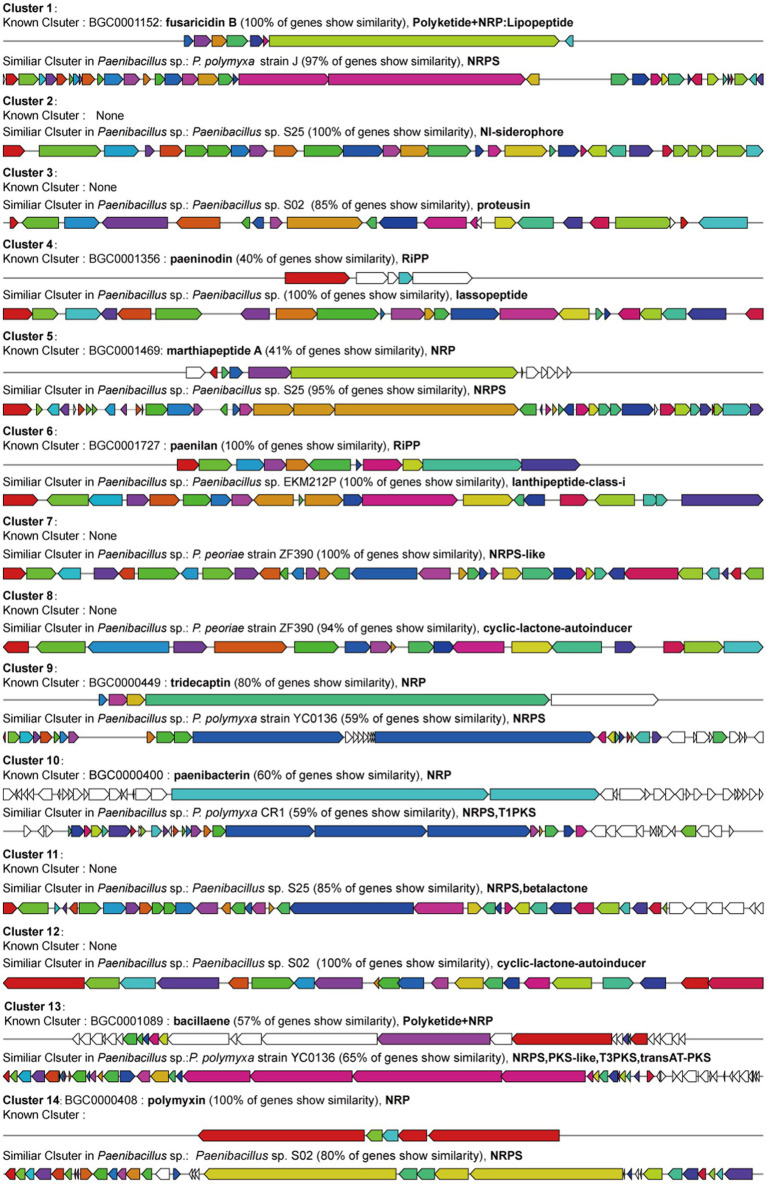
Prediction of secondary metabolites biosynthesis gene clusters in the strain YF identified by antiSMASH software. Of which, known clusters are similar gene clusters from MIBiG 3.1, and similar clusters are gene clusters identified in the *Paenibacillus* sp. genome rather than recruited in MIBiG datasets.

*Paenibacillus* species are also known to produce multiple types of antimicrobial peptides: ribosomally synthesized bacteriocins (ribosomally synthesized and post-translationally modified peptides, RiPPs), non-ribosomally synthesized peptides (NRPs), siderophore and polyketides (PKs) (Cochrane and Vederas, 2016; Grady et al., 2016). In order to further uncover antimicrobial peptides produced by YF, the genome was analyzed using the antiSMASH platform, an online tool for the prediction of secondary metabolite gene clusters. The analysis predicted 14 biosynthetic gene clusters (BGCs) related to secondary metabolites and the total length of BGCs accounting for 14.08% of the whole genome of the strain YF (Figure 6 and Supplementary Table S13). Of which, eight BGCs were similar to known clusters, and the clusters were identified in related *Paenibacillus* species, simultaneously. However, the other six BGCs were only similar to the clusters identified in related *Paenibacillus* species with high similarity. The polymyxins and fusaricidins were widely reported in *Paenibacillus* sp., and both of them are active against many important phytopathogens; the polymyxins B and fusaricidins B were identified in the genome of the strain YF, and both of them showed 100% of similarity with the known BGCs. Moreover, same as other *Paenibacillus* species, most of the antimicrobial peptides produced by the strain YF are synthesized non-ribosomally (e.g., marthiapeptide, tridecaptin, paenibacterin, betalactone, and paenibacterin), the non-ribosomal lipopeptides act primarily by disrupting membranes of the target cells (Ranjan et al., 2023). Among the remaining BGCs, two lasso peptide biosynthesis gene clusters (proteusin and paeninodin) and one lanthipeptide biosynthesis gene cluster (paenilan) were identified. These clusters belong to ribosomally synthesized and post-translationally modified peptides (RiPPs), specifically bacteriocins, which exhibit broad-spectrum antimicrobial activity against numerous pathogenic bacteria, including antibiotic-resistant strains (Subramanian and Smith, 2015). In addition, a gene cluster related to the biosynthesis of bacillaene was identified, which belongs to polyketides and inhibits prokaryotic growth by disrupting protein synthesis (Rath et al., 2010).

## 4 Discussion

Codonopsis root rot is considered one of the serious diseases affecting the harvest of codonopsis radix (Zhao et al., 2021; Zhang et al., 2023). The incidence of codonopsis root rot caused by *F. oxysporum* ranges from 20 to 70%, resulting in a significant decrease in codonopsis production, with a reduction rate of up to 60% (Zhang et al., 2023). Thus, the management of root rots is crucial for preventing codonopsis wilt and yield losses. This study investigated the biological control of *F. oxysporum* to suppress the codonopsis root rot by the antagonist strain *P. polymyxa* YF. The previous studies evaluated the biocontrol effect of *Paenibacillus* strains on various crops and found them to be effective against several fungal plant pathogens and diseases (Cochrane and Vederas, 2016; Grady et al., 2016), such as *Fusarium* root rot in Korean ginseng (Reeleder et al., 2002), sheath blight in maize (Chen et al., 2022), stem rot in cucumbers (Zhai et al., 2021), and bacterial leaf blight in rice (Abdallah et al., 2019), and also showed obviously growth-promoting effect in tomato (Mei et al., 2014) and wheat and many other plants (Li et al., 2023). Thus, there is no doubt that *Paenibacillus* sp. is one of the most effective BCA against plant pathogens and promotes plant growth. The strain YF was confirmed as *P. polymyxa* using a polyphasic approach, including phenotypic, physiological, biochemical characteristics and phylogenetic analysis based on 16S rRNA and whole-genome datasets, as well as average nucleotide identity (DDH and ANI). Of which, YF was clearly clustered with *P. polymyxa* and different from other *Paenibacillus* species in the 16S rRNA and whole genome-based dendrogram, simultaneously. Furthermore, with ANI values above 95% and DDH values above 70% indicating bacterial species boundaries, both results are congruent with the phylogenetic trees and support the classification of the strain YF as *P. polymyxa*.

*P. polymyxa* is widely recognized for its dual role as a biocontrol agent against a broad spectrum of phytopathogens and as a potent biofertilizer, as extensively documented in previous studies (Padda et al., 2017). As a strain belonging to *P. polymyxa*, the strain YF exhibited broad-spectrum antifungal activities, secreted indole-3-acetic acid (IAA), and showed significant promoting effect for codonopsis seedling growth, both of them will be the basis of successful application. In addition, bacterial colonization and biofilm formation on plant roots can also secure the colonization sites and serve as a nutrient sink in the rhizosphere, thereby diminishing the availability of root exudate-derived nutrients for pathogen stimulation or subsequent root colonization (Haggag and Timmusk, 2008; Høiby et al., 2010). The strain YF colonization on the root codonopsis reaches the highest colonization density 5 to 10 days after inoculation and further accomplishes the formation of biofilm, which also provides a valuable a feasible strategy for applying the strain YF in the cultivation of codonopsis.

Under the background that agricultural practice is currently moving from traditional chemical fertilizers and pesticides toward sustainable, effective, and environment-friendly agents, BCAs have emerged as an eco-smart tool for integrated disease management and growth promotion. In our study, the strain YF was compared with carbendazim treatment to investigate the antifungal activities of the strain YF, and a remarkable control efficacy against *F. oxysporum-induced* root rot *in vivo*, as the lower disease incidence, higher therapeutic efficiency, and the prevention effect of the strain YF even reach 100%, which further validate that the strain YF is a promising biological control agent to manage codonopsis root rot.

Furthermore, we identified the production of several hydrolytic enzymes in the strain YF, which have potential for biological control of phytopathogens and insect pests. These enzymes include chitinases, protease, cellulase, glucanase, and amylase. Of which, chitinases could cause lethal effects by hydrolyzing the chitin polymer in the fungal cell walls and insect cuticle (Bhattacharya et al., 2007); protease could hydrolyze the glycoprotein of fungal cell walls into small peptides and further lead cell lysis and cellular leakage, and the membranous layers of the insect exoskeleton, rich in structural proteins, serving as suitable targets for proteolytic activity (Ajuna et al., 2023); cellulose and *β*-glucans constitute the cell wall structure of fungal and oomycetes, respectively, and are an important target for biocontrol based on cell wall degradation strategies (Raza et al., 2008); and amylases contribute to the hydrolyze polysaccharides primarily in the fungal cell walls, enhancing the overall cell wall degrading activity of BCA against phytopathogenic fungal and bacterial diseases (Grady et al., 2016). Thus, strain YF exhibits promising potential for simultaneous biocontrol of fungal, bacterial, and pest-associated diseases, owing to its multi-targeted enzymatic activity against diverse pathogen structures. The inhibition effect and underlying mechanism of the strain YF to *F. oxysporum* were also further investigated, and the result revealed that YF significantly inhibited the secretion of fusaric acid, which is one of the major toxins of *F. oxysporum*.

Fungal virulence is a polyvalent, complex process that requires the expression of multiple genes at different stages and different sites of infection (Odds et al., 2001). Our results revealed that after confrontational culture of YF, multiple genes that were related to the whole dynamic process of pathogens invading plants, including infection, colonization, pathogenicity, and hyphal development, were downregulated in *F. oxysporum*, indicating that fungal virulence genes as targets for the strain YF.

To elucidate the molecular underpinnings of the biocontrol and plant growth-promoting mechanisms of *P. polymyxa* YF, the whole genome was completely sequenced and assembled. Genome mining analyses were also conducted, and numerous genes related to potential bacterial molecular processes of biofertilizers and biocontrol agents were identified. The *Ipd*C gene that encodes key enzymes in the IAA biosynthesis and other eight genes related to the biosynthesis of precursor for IAA have been identified in the YF genome, correspondingly. In addition, *Pseudomonads* spp. are also believed to be an effective PGPR due to their capacity to fix nitrogen, solubilize mineral phosphates, transport potassium, utilize magnesium, increase plant tolerance to abiotic stress by trigger ISR and PTI through emission of VOC, and other good traits (Raza et al., 2008; Eastman et al., 2014; Grady et al., 2016). Based on genome mining, the genome of YF contains the *Nif* gene cluster to facilitate the conversion of atmospheric N_2_ into bioavailable NH_3,_ and the genes related to potassium and magnesium utilization were also detected. Additionally, the genes involved in encoding elicitors (e.g., 2,3-butanediol and carboxypeptidase) that could induce plant systemic resistance and pathogen or microbe-triggered immunity were identified in the YF genome. Hence, the strain YF with its multifunctional ability to promote plant growth, indicates great potential as an effective biofertilizer in ecological and commercial agriculture.

*Paenibacilli* sp. are efficient producers of potent metabolites against bacterial and fungal pathogens, which are of great interest in agriculture, medicine, and food processing (Eastman et al., 2014; Grady et al., 2016). By using antiSMASH, a total of 14 secondary metabolites BGCs were detected in the YF genome. Among these, polymyxins and fusaricidin were originally identified in *P. polymyxa,* and exhibitted distinct antimicrobial profiles: polymyxins demonstrate potent activity against Gram-negative bacteria, while fusaricidin displays broad-spectrum antifungal efficacy against numerous phytopathogenic fungi (Mülner et al., 2021); paenibacterin disrupts the cell membrane integrity of fungal pathogens (Huang and Yousef, 2014); paenilan (Park et al., 2017) and tridecaptin (Bakhtiary, 2018) with potent activity selectively target Gram-negative bacteria; paeninodin provides a broad variety of antimicrobial activities; betalactone (Wackett, 2017) inhibits the central virulence regulator of bacterial pathogens; bacillaene (Miao et al., 2024) showed potent antifungal activity and was first reported in *Bacillus subtilis* as it enhanced the biocontrol efficiency of it; marthiapeptide (Zhou et al., 2012) also is an anti-infective and cytotoxic cyclic peptide. Overall, YF the powerful abilities in plant growth-promoting and is regarded as an agriculturally important microorganism. Concurrently, it is capable of producing a variety of antibiotic compounds and antifungal compounds and effectively inhibiting the growth of pathogens under both laboratory and field conditions. In particular, the copious secondary metabolite BGCs in the genome of *P. polymyxa* YF likely confer the attributes of a promising BCA, exhibiting broad-spectrum and efficient antagonistic activity against fungal plant pathogens.

## 5 Conclusion

In our present study, a *P. polymyxa* YF isolated from sheep manure compost of healthy codonopsis exhibited broad-spectrum antibacterial activities against *F. oxysporum*. This strain exhibited the ability to form biofilms and colonize codonopsis roots, effectively managing codonopsis root rot induced by *F. oxysporum*. The inhibited effects of the strain YF for the pathogenicity of *F. oxysporum* were verified at both metabolic and transcriptional levels. Genomic mining revealed that the strain YF possesses multiple genes associated with indole-3-acetic acid (IAA) production, nitrogen fixation, phosphate solubilization, antifungal activity, and resistance inducer biosynthesis. Additionally, several key BGCs were predicted indicating the ability of stain YF products various bioactive metabolites, such as polymyxins, fusaricidin, paenibacterin, paenilan, paeninodin, betalactone, and bacillaene. Consequently, the strain YF emerges as a promising biocontrol agent to inhibit root rot induced by pathogen *F. oxysporum* and a biofertilizer promotes the growth of codonopsis.

## Data Availability

The genome assembly file and genomic sequencing data have been deposited at NCBI under the BioProject accession numbers of PRJNA1215726. All other data are available from the corresponding authors on reasonable request.

## Footnotes

^1^ https://automlst.ziemertlab.com/
