# Supplementary material for: Genomic characterization and antifungal properties of Paenibacillus polymyxa YF, a promising biocontrol agent against Fusarium oxysporum pathogen of codonopsis root rot
Source: Front Microbiol. 2025 Feb 26;16:1549944. doi: 10.3389/fmicb.2025.1549944 (PMC11897986; doi:10.3389/fmicb.2025.1549944)
Supplement: Supplementary file 1 [file Data_Sheet_1.docx]

**­**

# Supplementary Materials for

**Genomic characterization and antifungal mechanisms of *Paenibacillus polymyxa* YF, a promising biocontrol agent against *Fusarium oxysporum* pathogen of codonopsis root rot**

The file includes:

Supplementary Figures. 1 to 16

Supplementary Tables 1 to 11


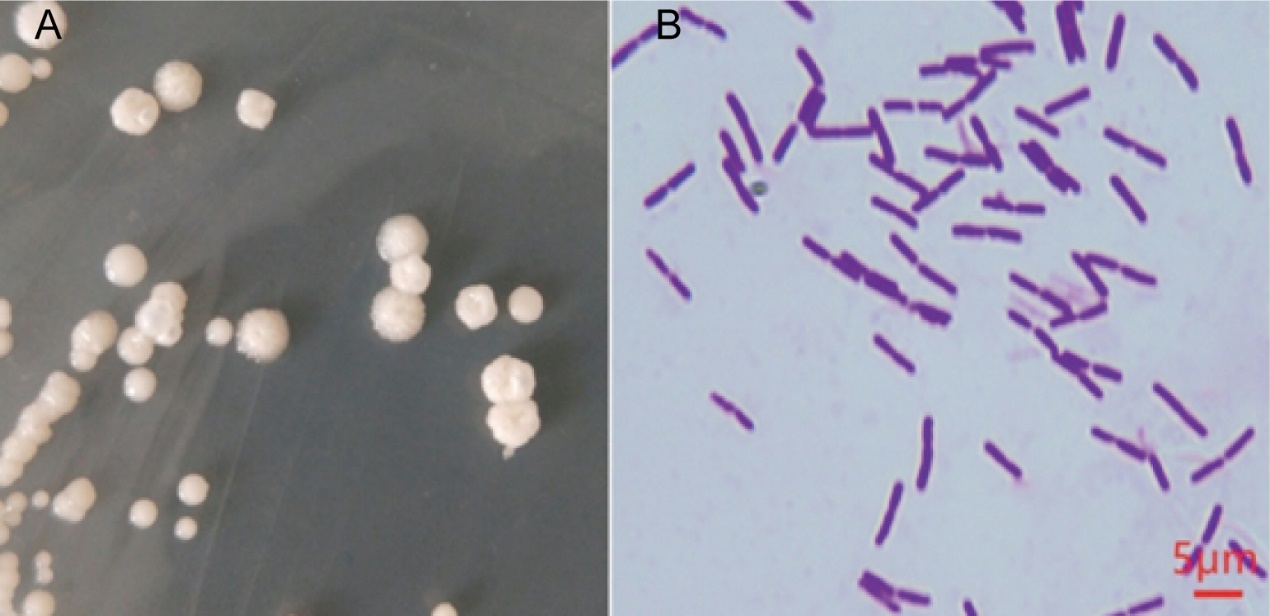


**Fig. S1.** Colony morphology (A) and **Gram staining (B) of strain YF.**


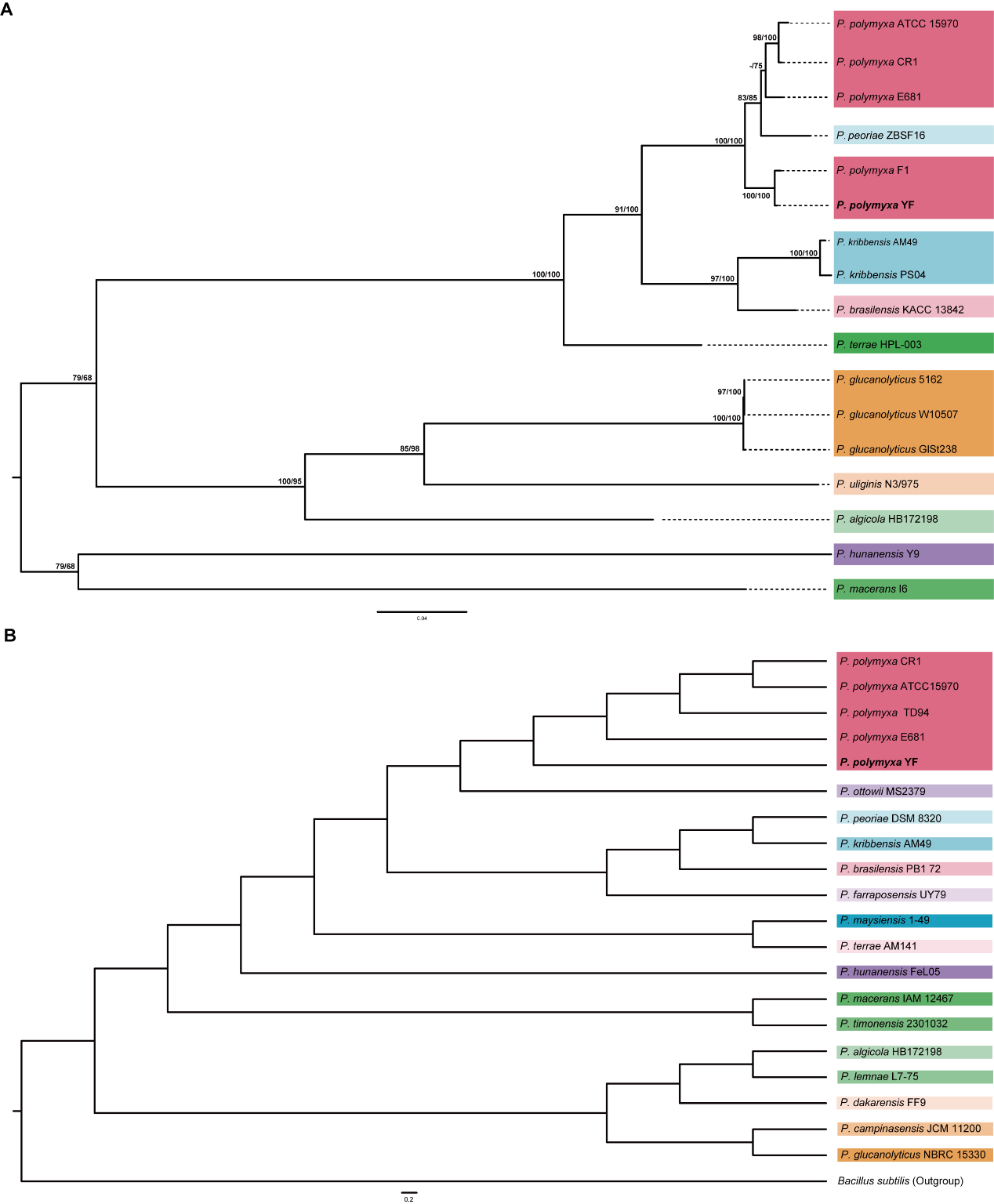


**Fig. S2. Phylogenetic tree based on a housekeeping gene *gryB* (A) and 81 conserved genes (B) of** *Paenibacillus* species that identified by autoMLST**.**


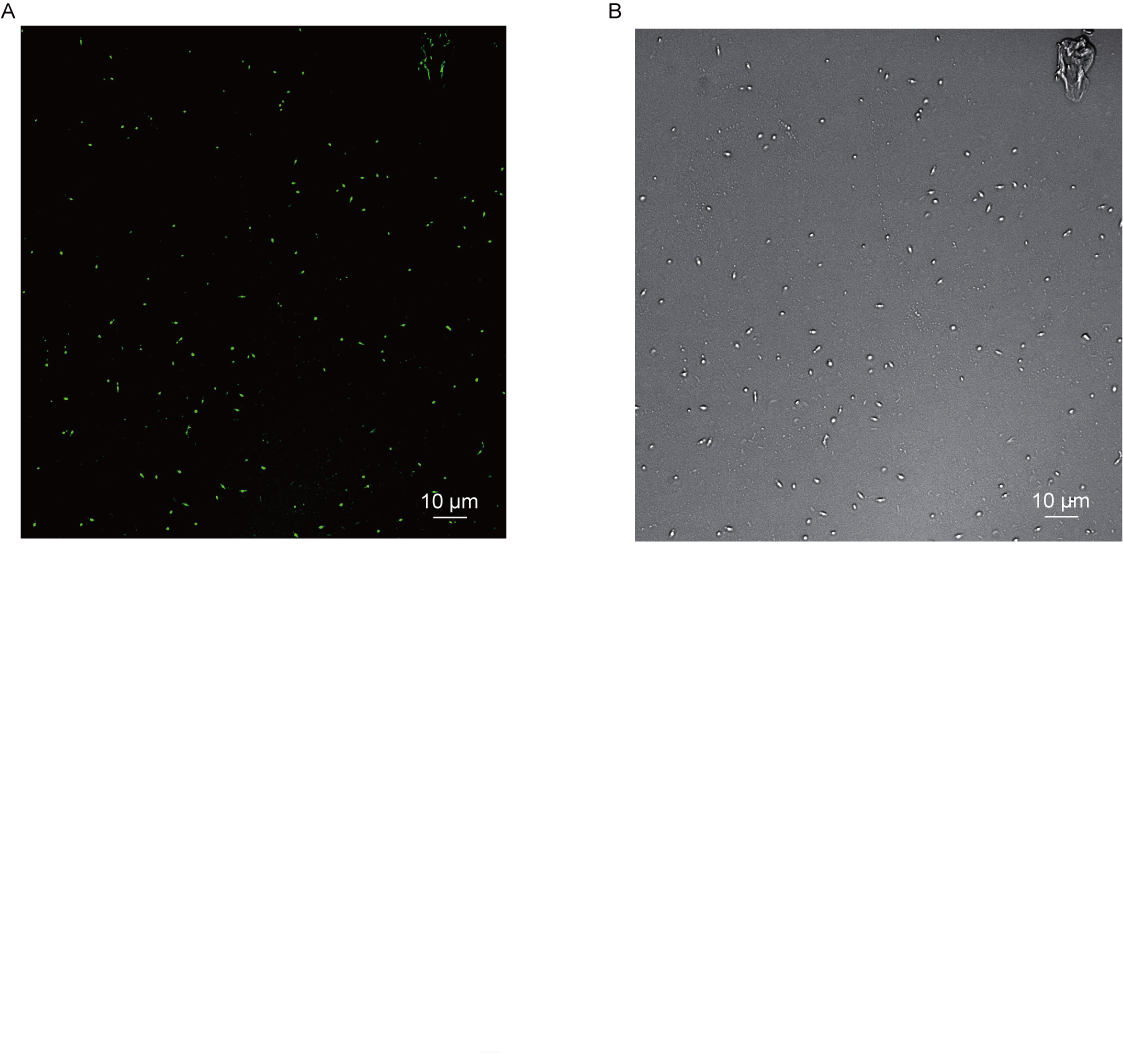


**Fig. S3.** pGFP4412 expression in YF strain (40× magnification using fluorescence microscopy) (A:485 nm Blue excited light; B: natural light).


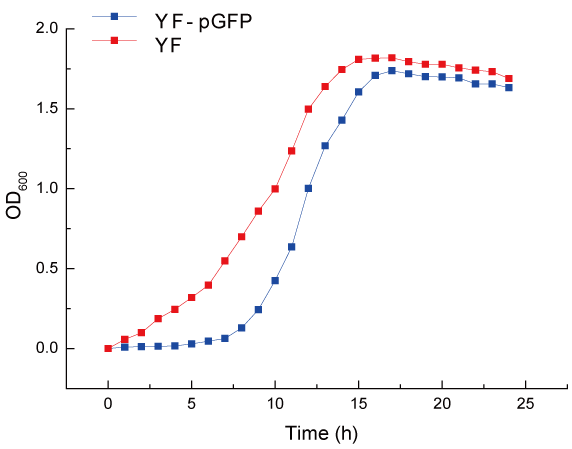


**Fig. S4.** Growth curves of strains YF and YF – pGFP.


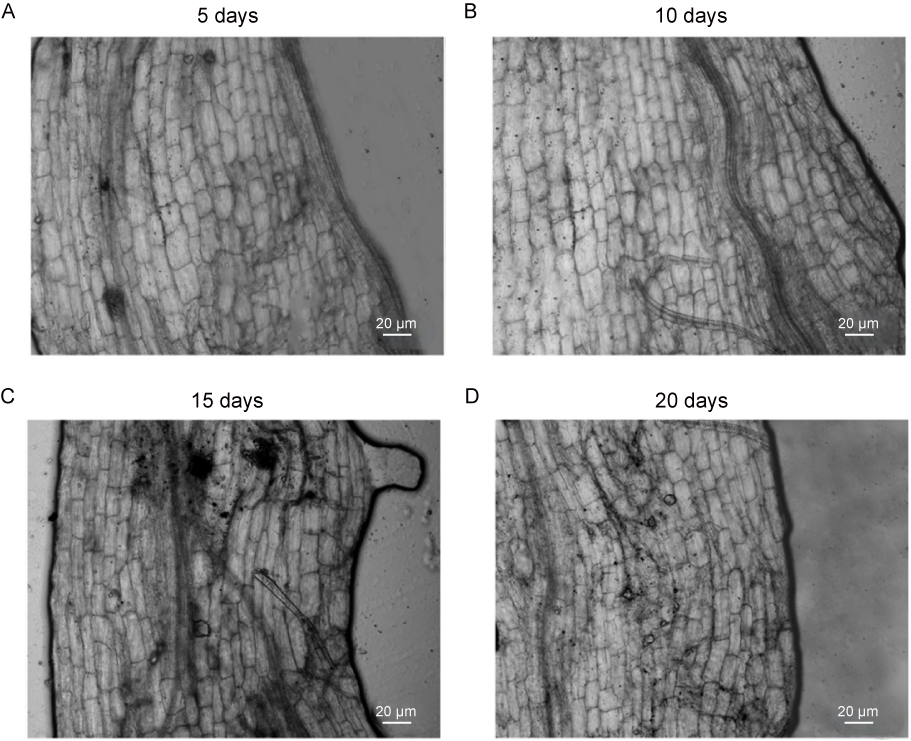


**Fig. S5. Colonization of strain YF-pGFP in the roots of codonopsis for 5 days (A), 10 days (B), and 15 days (C) and 20 days (D), respectively.** GFP fluorescence exhibits excitation at nature light.


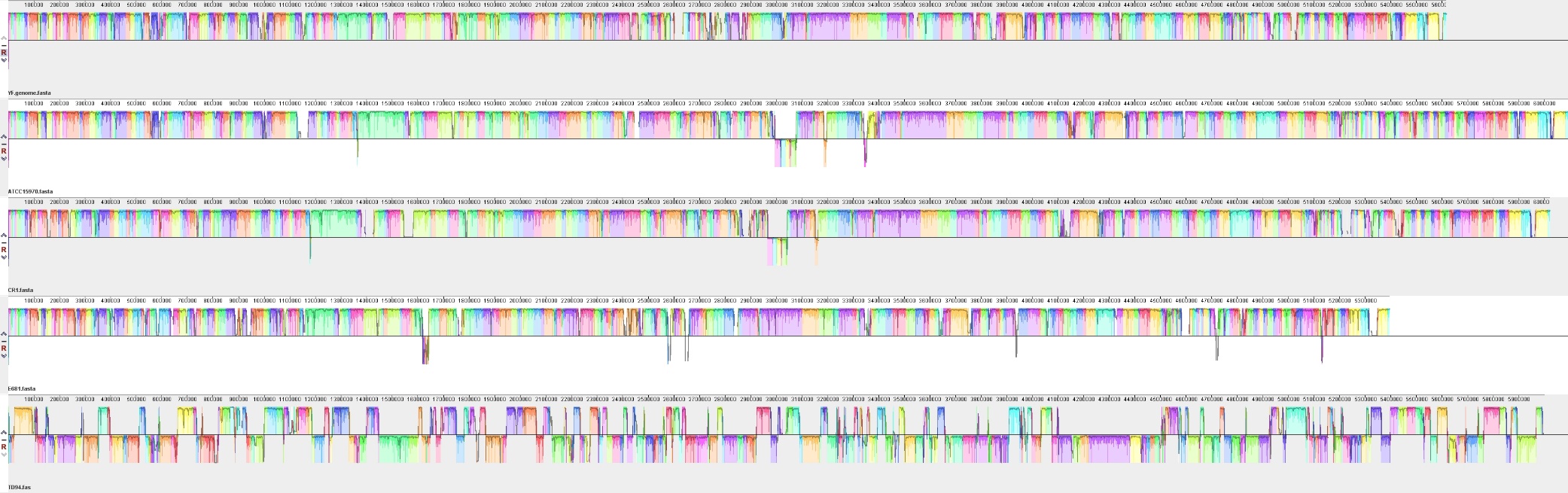


*P. polymyxa* ATCC15970

*P. polymyxa* CR1

*P. polymyxa* E681

*P. polymyxa* TD94

*P. polymyxa* YF

**Fig. S6. Genome-to-genome alignment of different strain of *P. polymyxa* using a progressive mauve software with a window of 1,000 nucleotides and YF as the reference genome.** Boxes with the same color indicate the syntenic regions. Boxes below the horizontal line indicate inverted regions.


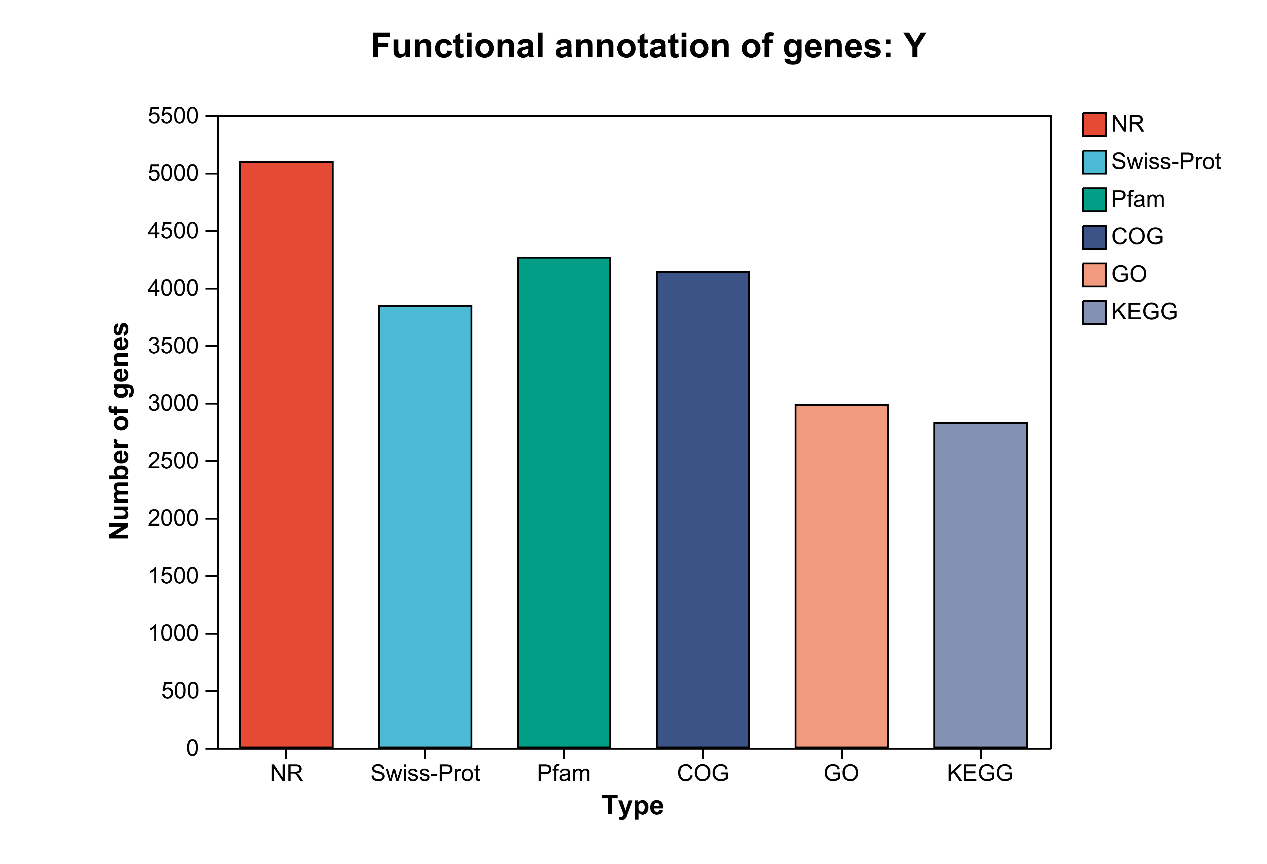


**Fig. S7. Statistics for functional annotation of protein coding genes.**


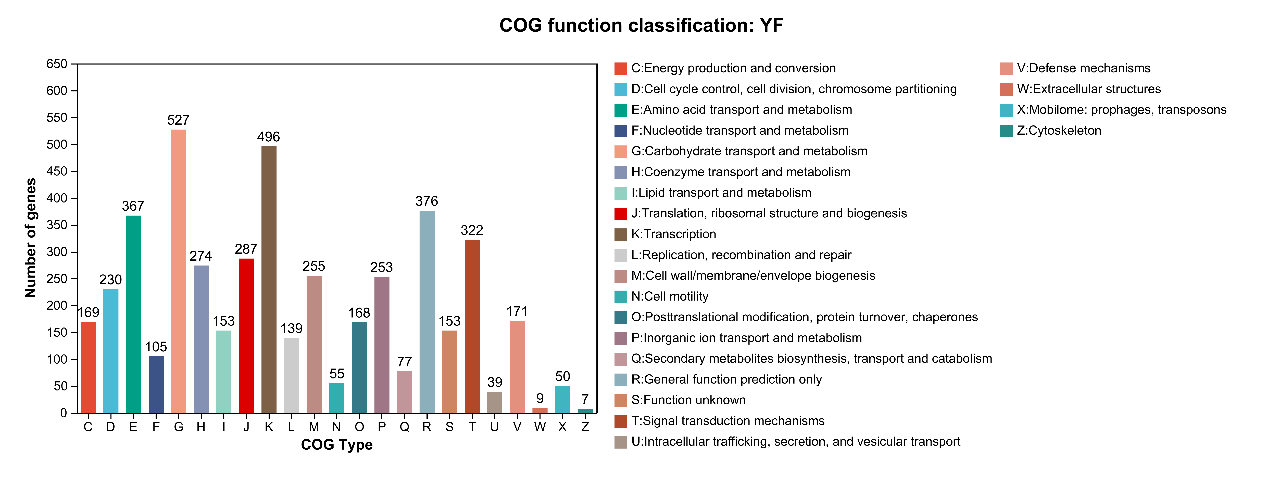


**Fig. S8. Distribution of genes across COG functional categories in the genome of YF.**


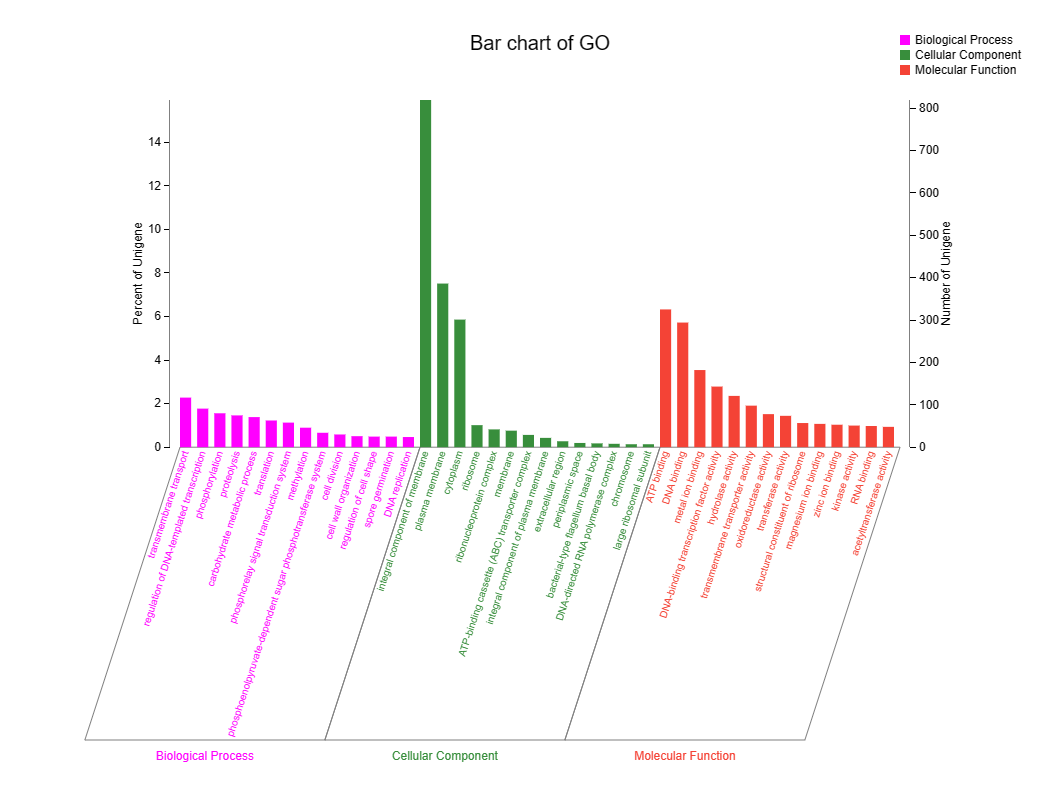


**Fig. S9.** **Biological process, cellular component, and molecular function terms of YF genome annotated by using the Gene Ontology database.**


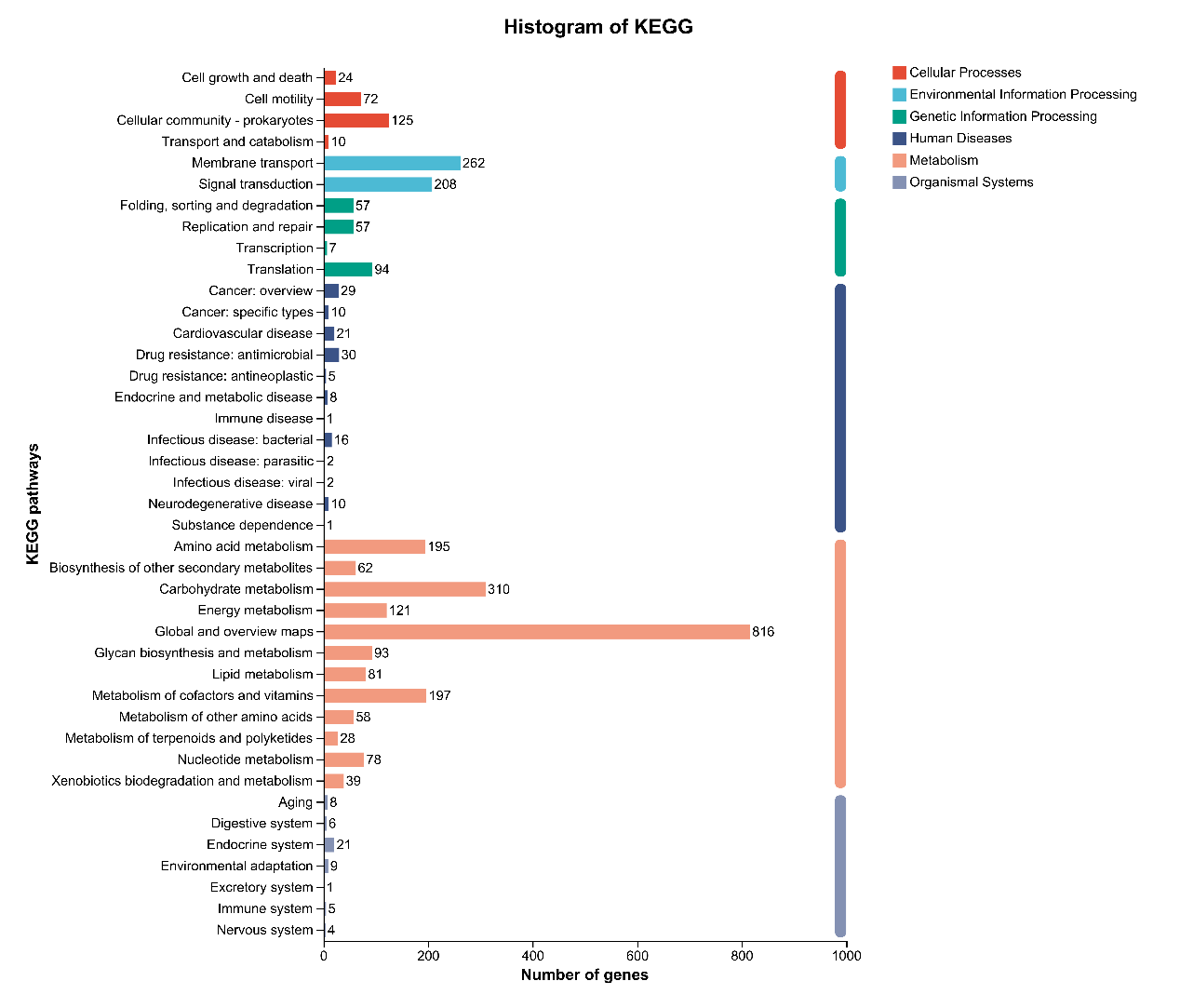


**Fig. S10. Metabolic pathways of YF genome annotated by using the Kyoto Encyclopedia of Genes and Genomes (KEGG) database.**


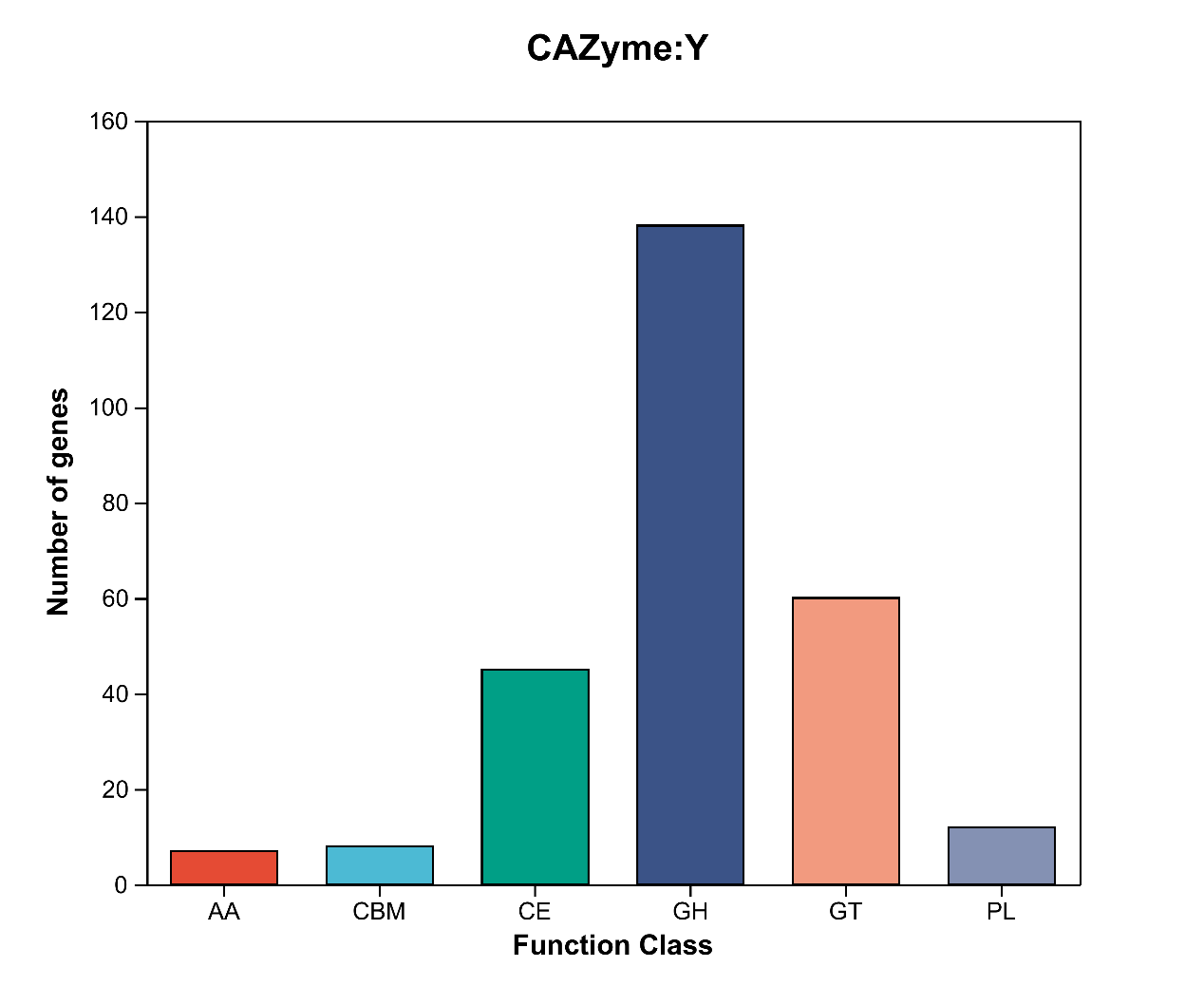


**Fig. S11.** **Determination of biological activity of YF:** Auxiliary Activities (AA); Carbohydrate-Binding Modules (CBM); Carbohydrate Esterases (CE); Glycoside Hydrolases (GH); Glycosyl Transferases (GT); Polysaccharide Lyases (PL).


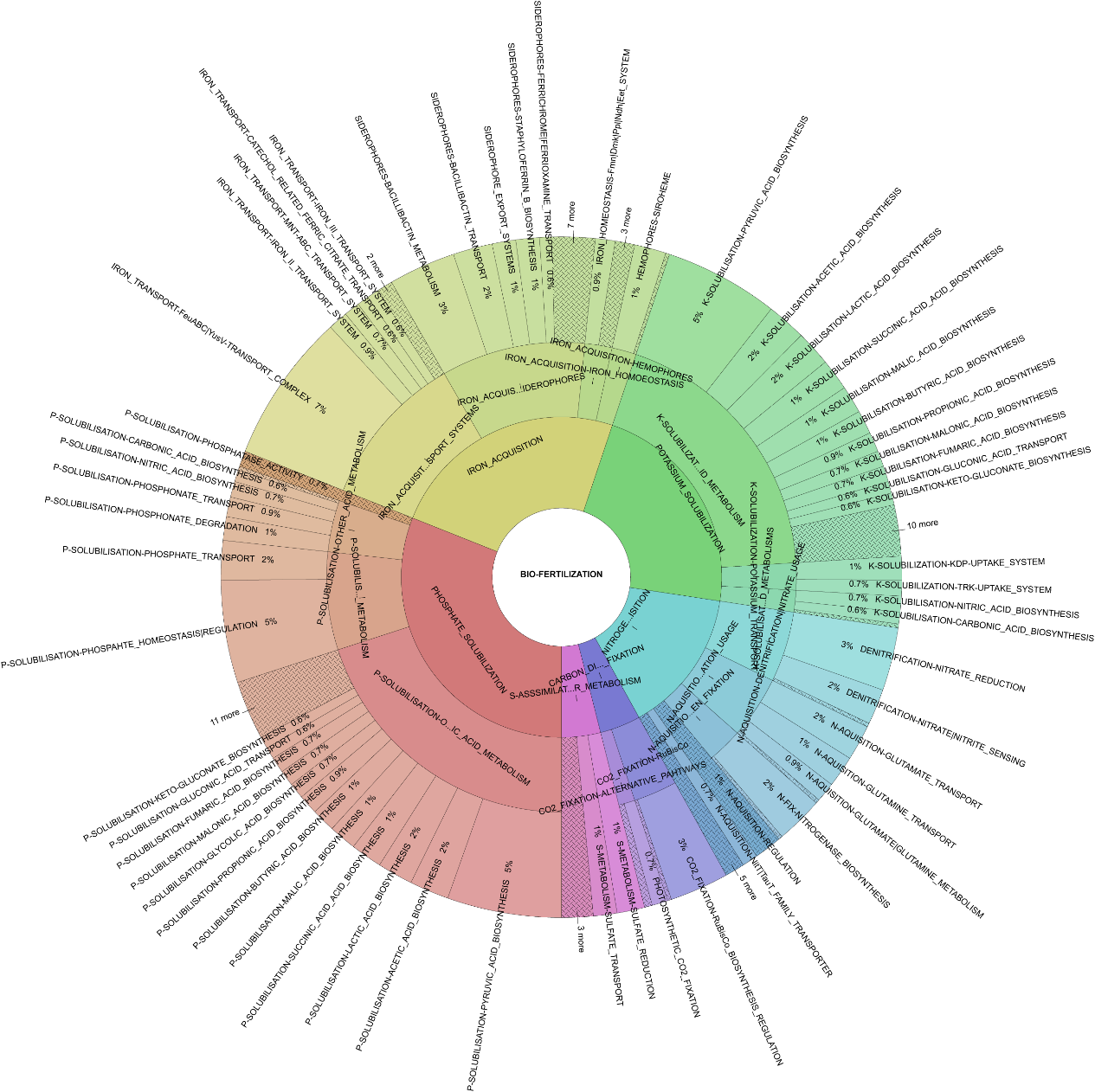


**Fig. S12. Krona plot of bio-fertilazation genes observed in the genome of strain YF.**


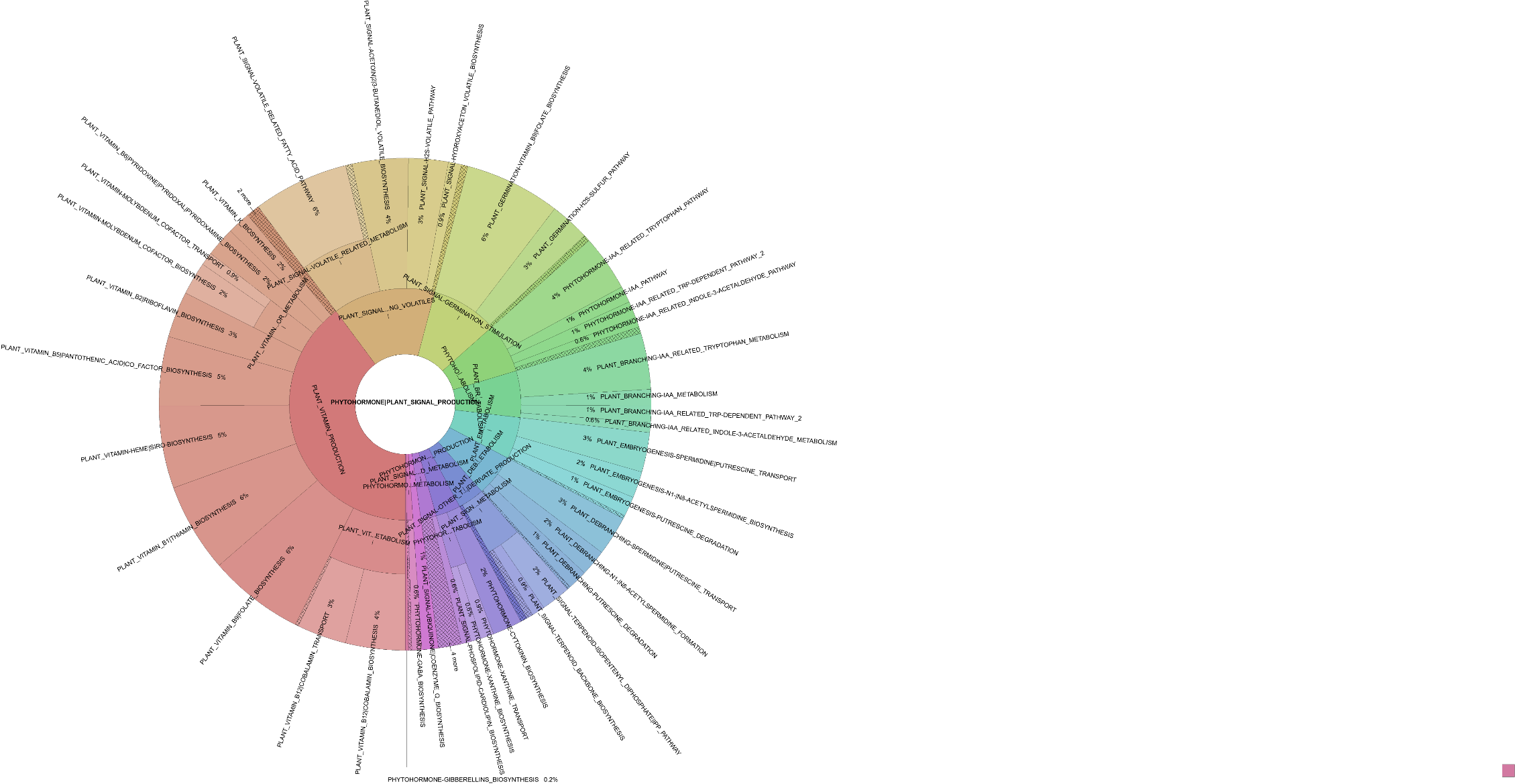


**Fig. S13. Krona plot of phytohormones genes observed in the genome of strain YF.**


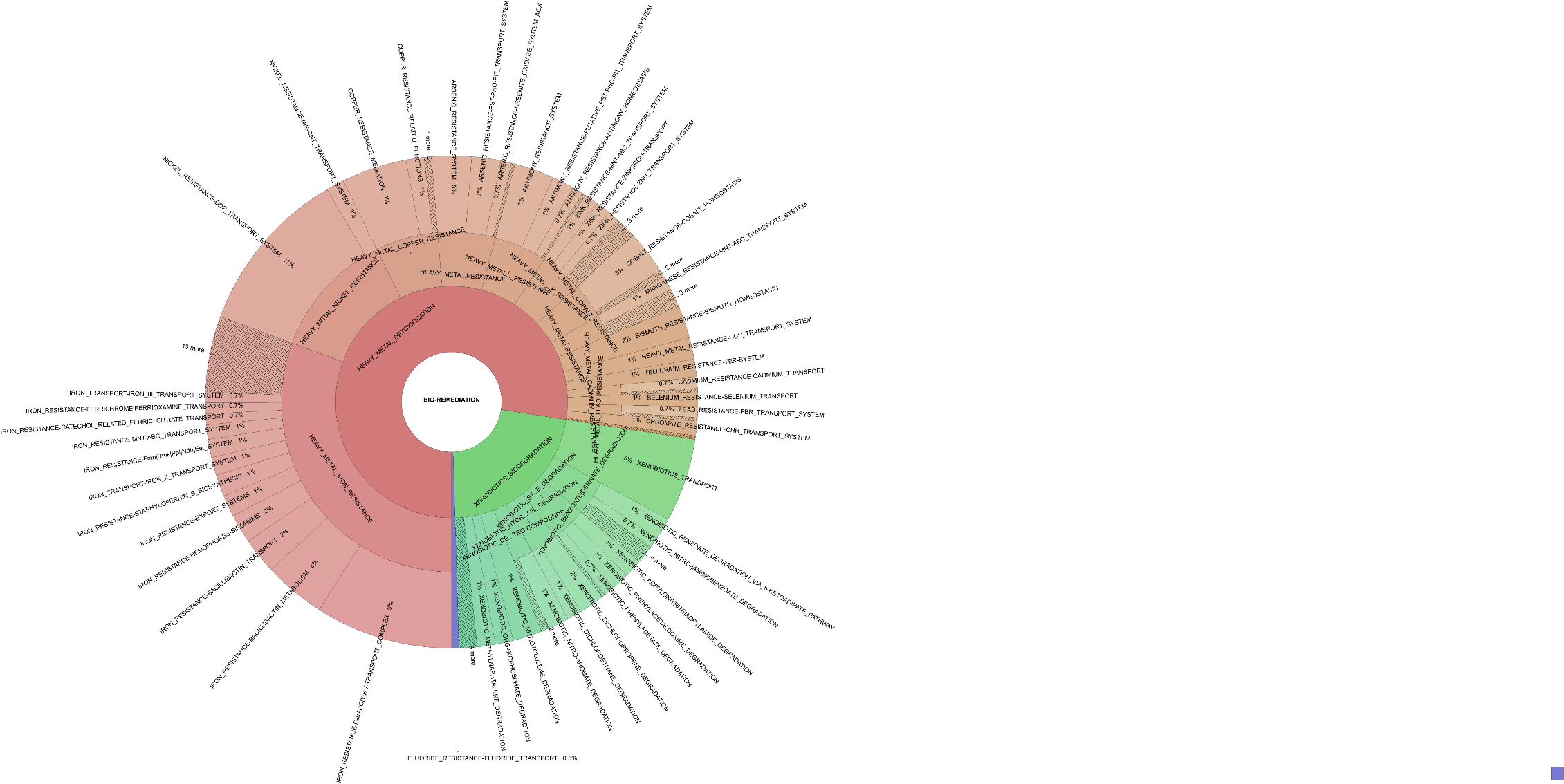


**Fig. S14. Krona plot of bio-remediation genes observed in the genome of strain YF.**


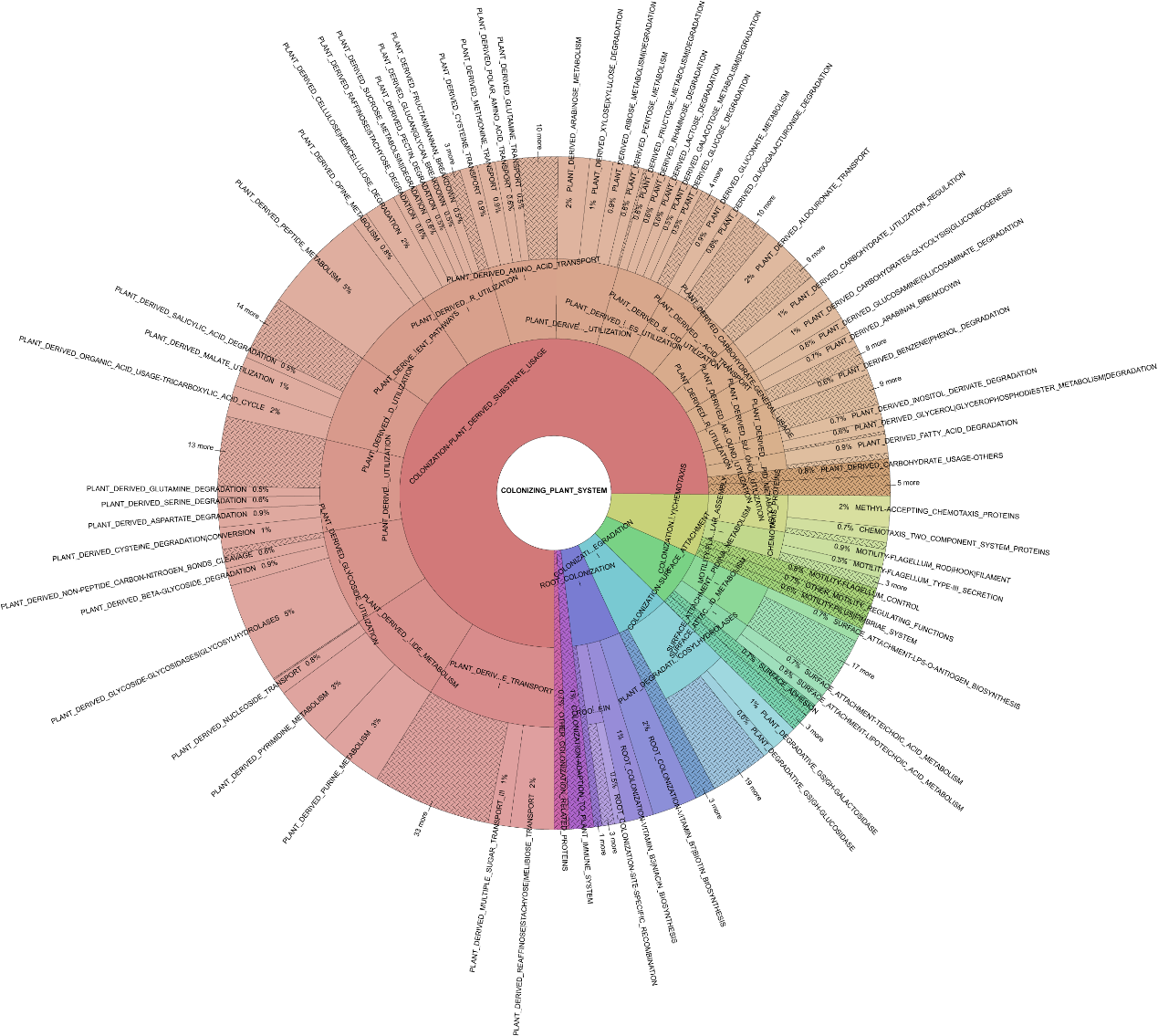


**Fig. S15. Krona plot of colonizing plant system genes observed in the genome of strain YF.**


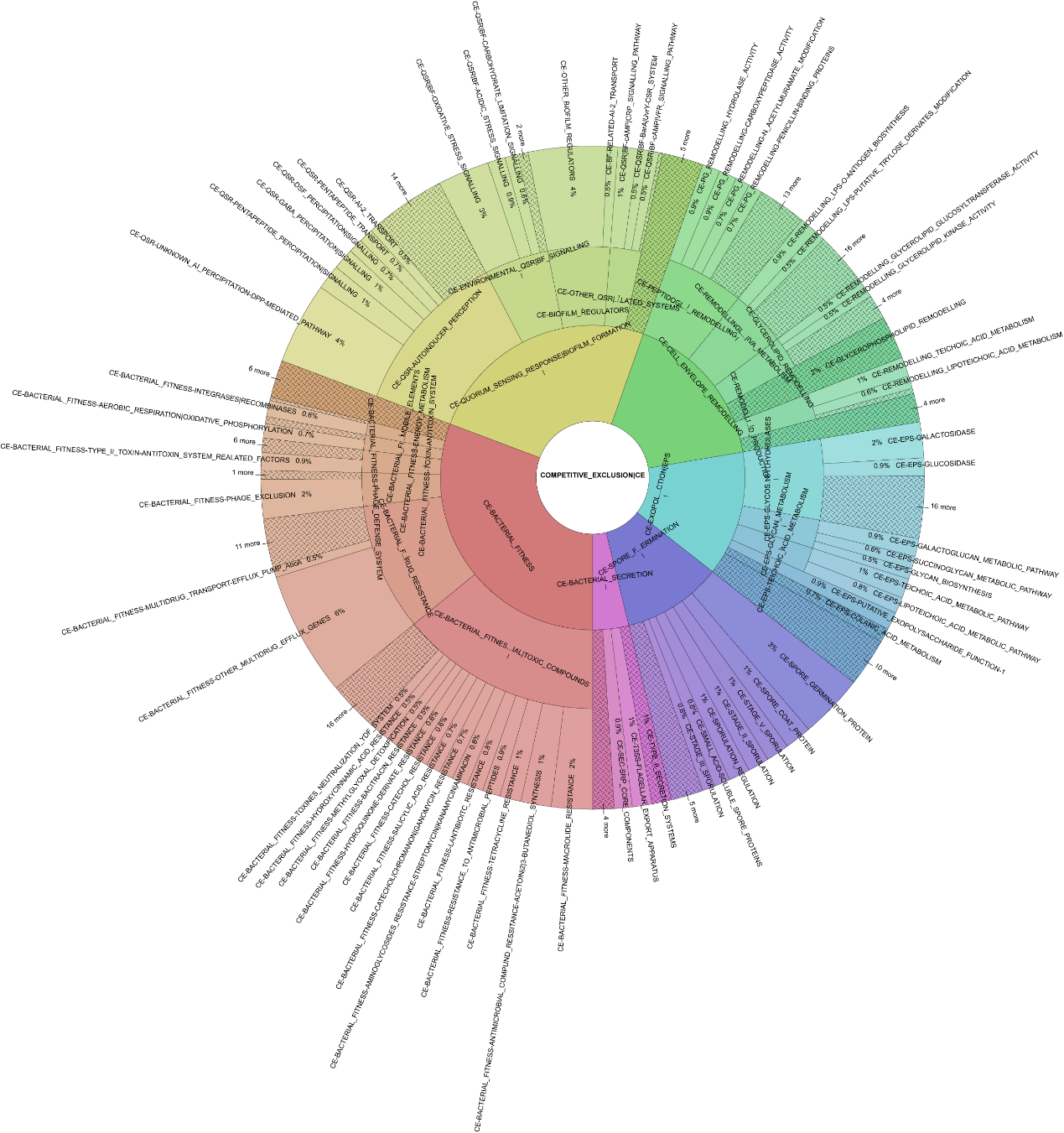


**Fig. S16. Krona plot of** **competitive exclusion genes observed in the genome of strain YF.**


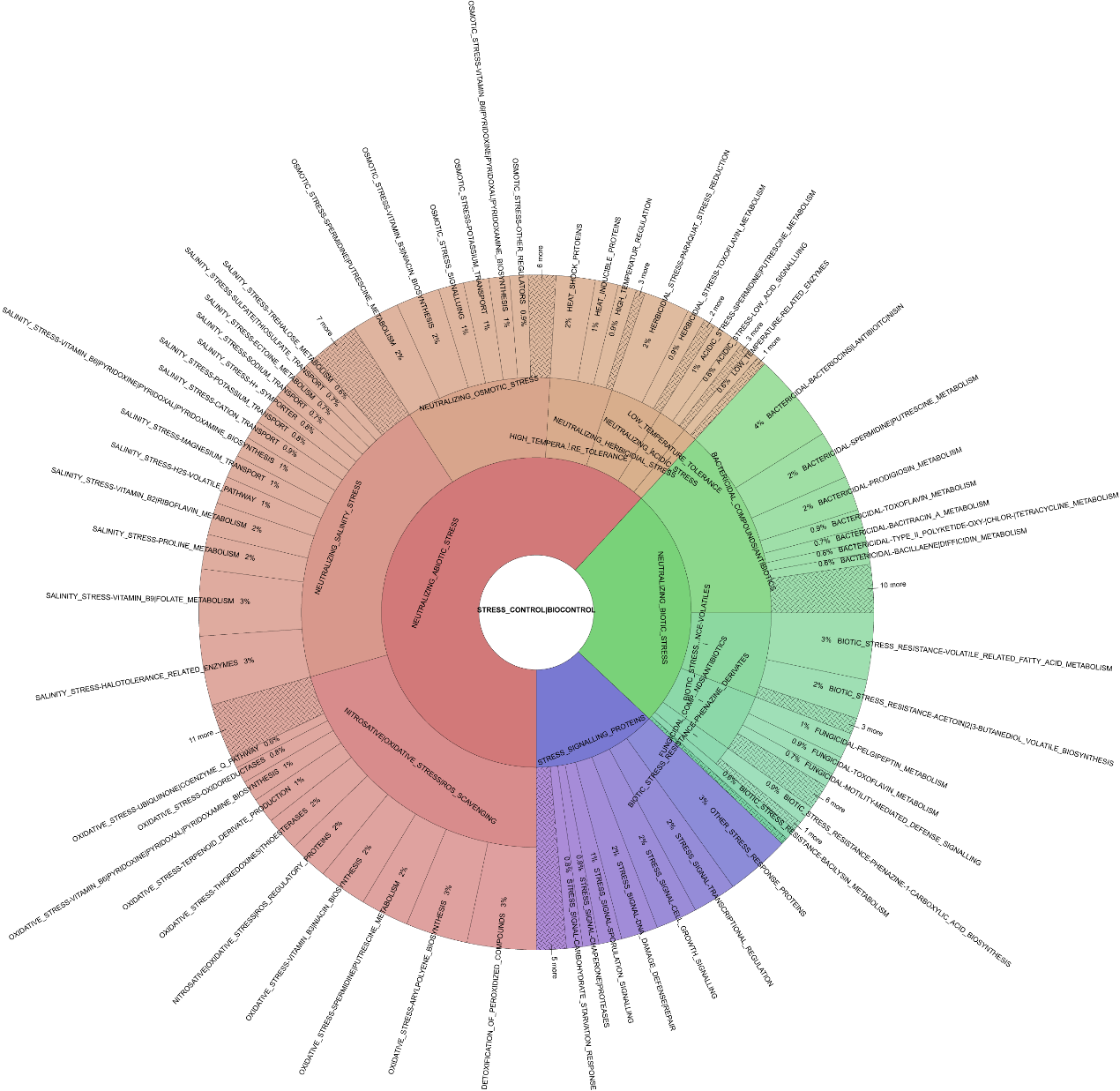


**Fig. S17. Krona plot of stress control genes observed in the genome of strain YF.**

**­Table S1.** *F. oxysporium* disease-associated gene qRT-PCR primer sequence.

| Gene name | Primer sequence (5' to 3') |
| --- | --- |
| PL1 | AGTACACTGCCATCCTCGCC |
|  | GCAGCTCGTGGTAACTCCA |
| PG1 | GCCCGACCATTTCATCGTTG |
|  | GGCACCAGAGGGAATTCCCT |
| PGX1 | ATAACGTTCTTTCGGCGGTC |
|  | ATTGGGCGGTTTCTCATTCG |
| fpd1 | GTTCACGCCAAACACCCAATC |
|  | ATCCACTGCCTGCTCCACAAG |
| SIX8 | ATGTTGGACCCTCCCTCTATT |
|  | CTACTACCAATTGTTTATAAACTG |
| SIX1 | GCGCTTCATGATCTGTGCGTTGAA |
|  | ATCGCAACTTAGTGTGGGCTGGTA |
| Fmk1 | AGCGACTGCGCATTCTTCTCTACA |
|  | TGAGATCACAGTTGGCGTTGAGGA |
| SNF1 | TTTCTCAAGACAAGCTGTGGCAGTCCC |
|  | TGTTCTGTTGCTGGGAGGTTCGTCG |
| PelD | GTGAAGTGCTCTGGTCAGGCTGAGG |
|  | TGGACTTGTCGCAGCCCTTGTAACC |
| FEM1 | AAAGAATTCACCTCCGCCACTGGTGACTC |
|  | AAATCTAGACCGCTCTCAGGGACACTGG |
| Rho1 | GAAAGCTCGTCATTGTCGGCGATGG |
|  | CTCGATGGTCTTCTGGTCGTATCGC |

**Table S2.** The accession numbers of the strains used for the genomic phylogeny analysis.

| **Number** | **Species** | | **Srain** | | **Genbank ID** |
| --- | --- | --- | --- | --- | --- |
| 1 | *Paenibacillus polymyxa* | CR1 | | NC_023037.2 | |
| 2 | *Paenibacillus polymyxa* | E681 | | NC_014483.2 | |
| 3 | *Paenibacillus polymyxa* | ATCC15970 | | NZ_CP011420.1 | |
| 4 | *Paenibacillus polymyxa* | TD94 | | GCA_000520775.1 | |
| 5 | *Paenibacillus algicola* | HB172198 | | CP040396.1 | |
| 6 | *Paenibacillus brasilensis* | DSM 1491 | | ASM3081498v1 | |
| 7 | *Paenibacillus campinasensis* | 7537-G1 | | GCF_002272015 | |
| 8 | *Paenibacillus dakarensis* | FF9 | | CDSE01000001 | |
| 9 | *Paenibacillus farraposensis* | UY79 | | GCA_020736845.1 | |
| 10 | *Paenibacillus glucanolyticus* | NBRC 15330 | | GCA_004000885.1 | |
| 11 | *Paenibacillus hunanensis* | DSM 22170 | | GCA_031454175.1 | |
| 12 | *Paenibacillus kribbensis* | PS04 | | GCA_013394225.1 | |
| 13 | *Paenibacillus lemnae* | L7-75 | | GCA_012933385.1 | |
| 14 | *Paenibacillus macerans* | NCTC6355 | | GCA_900454495.1 | |
| 15 | *Paenibacillus maysiensis* | 1-49 | | GCA_000520815.1 | |
| 16 | *Paenibacillus ottowii* | MS2379 | | GCA_006874425.1 | |
| 17 | *Paenibacillus terrae* | HPL-003 | | GCA_000235585.1 | |
| 18 | *Paenibacillus timonensis* | DSM 16943 | | GCA_022427145.1 | |
| 19 | *Paenibacillus peoriae* | KCTC 3763 | | GCA_000236805.2 | |

**Table S3.** Orthogonal array results of YF fermentation media.

| **Medium** | **Sucrose** | | **Peptone** | | **Magnesium sulfate** | **Sodium chloride** | | **OD_600_** |
| --- | --- | --- | --- | --- | --- | --- | --- | --- |
| 1 | 1(5) | 1(5) | | 1(3) | | 1(3) | 0.587±0.006 | |
| 2 | 1(5) | 2(10) | | 2(5) | | 2(5) | 0.613±0.005D | |
| 3 | 1(5) | 3(15) | | 3(7) | | 3(7) | 0.697±0.007 | |
| 4 | 2(10) | 1(5) | | 2(5) | | 3(7) | 0.601±0.005 | |
| 5 | 2(10) | 2(10) | | 3(7) | | 1(3) | 0.688±0.005 | |
| 6 | 2(10) | 3(15) | | 1(3) | | 2(5) | 0.698±0.004 | |
| 7 | 3(15) | 1(5) | | 3(7) | | 2(5) | 0.787±0.005 | |
| 8 | 3(15) | 2(10) | | 1(3) | | 3(7) | 0.634±0.006 | |
| 9 | 3(15) | 3(15) | | 2(5) | | 1(3) | 0.883±0.005 | |
| K_1_ | 0.616 | 0.631 | | 0.639 | | 0.704 |  | |
| K_2_ | 0.655 | 0.665 | | 0.713 | | 0.668 |  | |
| K_3_ | 0.759 | 0.729 | | 0.672 | | 0.660 |  | |
| R | 0.163 | 0.089 | | 0.074 | | 0.049 |  | |

**Table S4.** Partial 16S rRNA sequence alignment results from NCBI BLAST searches for strain YF.

| **Strain** | **Max Score** | **Total Score** | **Query Cover (%)** | **Per. Ident (%)** | **Acc. Len (bp)** | **Accession** |
| --- | --- | --- | --- | --- | --- | --- |
| *P. polymyxa* strain DSM 36 | 2532 | 2532 | 99 | 98.87 | 1547 | NR_117732.2 |
| *P. peoriae* strain 3763 | 2527 | 2527 | 99 | 98.8 | 1501 | [NR_042092.1](https://www.ncbi.nlm.nih.gov/nucleotide/NR_042092.1?report=genbank&log$=nucltop&blast_rank=2&RID=1Y9076N3013) |
| *P. farraposensis* strain UY79 | 2390 | 2390 | 94 | 98.66 | 1348 | NR_181417.1 |
| *P. peoriae* KCTC 3763 strain DSM 8320 | 2512 | 2512 | 99 | 98.59 | 1541 | [NR_117743.1](https://www.ncbi.nlm.nih.gov/nucleotide/NR_117743.1?report=genbank&log$=nucltop&blast_rank=6&RID=1Y9076N3013) |
| *P. polymyxa* strain IAM 13419 | 2518 | 2518 | 99 | 98.59 | 1504 | NR_112117.1 |
| *P. kribbensis* strain AM49 | 2479 | 2479 | 99 | 98.18 | 1511 | [NR_025169.1](https://www.ncbi.nlm.nih.gov/nucleotide/NR_025169.1?report=genbank&log$=nucltop&blast_rank=25&RID=1Y9076N3013) |
| *P. ottowii* strain MS2379 | 2471 | 2471 | 99 | 98.1 | 1476 | NR_180200.1 |
| *P. brasilensis* strain PB1 72 | 2353 | 2353 | 94 | 98.01 | 1384 | [NR_025106.1](https://www.ncbi.nlm.nih.gov/nucleotide/NR_025106.1?report=genbank&log$=nucltop&blast_rank=30&RID=1Y9076N3013) |
| *P. maysiensis* strain 1-49 | 2446 | 2446 | 99 | 97.75 | 1440 | [NR_165764.1](https://www.ncbi.nlm.nih.gov/nucleotide/NR_165764.1?report=genbank&log$=nucltop&blast_rank=33&RID=1Y9076N3013) |
| *P. terrae* strain AM141 | 2436 | 2436 | 99 | 97.62 | 1513 | [NR_025170.1](https://www.ncbi.nlm.nih.gov/nucleotide/NR_025170.1?report=genbank&log$=nucltop&blast_rank=34&RID=1Y9076N3013) |
| *P. triticisoli* strain BJ-18 | 2420 | 2420 | 99 | 97.23 | 1480 | [NR_178503.1](https://www.ncbi.nlm.nih.gov/nucleotide/NR_178503.1?report=genbank&log$=nucltop&blast_rank=35&RID=1Y9076N3013) |
| *P. peoriae* strain IFO 15541 | 2346 | 2346 | 96 | 96.69 | 1437 | [NR_115601.1](https://www.ncbi.nlm.nih.gov/nucleotide/NR_115601.1?report=genbank&log$=nucltop&blast_rank=36&RID=1Y9076N3013) |
| *P. dakarensis* strain FF9 | 2200 | 2200 | 96 | 95.52 | 1517 | NR_169361.1 |
| *P. campinasensis* strain 324 | 2183 | 2183 | 96 | 95.29 | 1508 | NR_024857.1 |
| *P. campinasensis* strain JCM 11200 | 2191 | 2191 | 96 | 95.29 | 1503 | NR_112162.1 |
| *P. glucanolyticus* strain NBRC 15330 | 2176 | 2176 | 96 | 95.17 | 1481 | NR_113748.1 |
| *P. timonensis* 2301032 | 2233 | 2233 | 99 | 95.08 | 1476 | NR_115199.1 |
| *P. terreus* strain D33 | 2231 | 2231 | 99 | 95.08 | 1516 | [NR_147741.1](https://www.ncbi.nlm.nih.gov/nucleotide/NR_147741.1?report=genbank&log$=nucltop&blast_rank=42&RID=1Y9076N3013) |
| *P. glucanolyticus* strain DSM 5162 | 2167 | 2167 | 96 | 95.02 | 1535 | NR_040883.1 |
| *P. hunanensis* strain FeL05 | 2228 | 2228 | 99 | 95.01 | 1514 | NR_116440.1 |
| *P. algicola* strain HB172198 | 2218 | 2218 | 99 | 94.88 | 1477 | NR_179981.1 |
| *P. macerans* strain IAM 12467 | 2158 | 2158 | 96 | 94.86 | 1504 | [NR_040886.1](https://www.ncbi.nlm.nih.gov/nucleotide/NR_040886.1?report=genbank&log$=nucltop&blast_rank=46&RID=1Y9076N3013) |
| *P. provencensis* strain 4401170 | 2213 | 2213 | 99 | 94.81 | 1457 | NR_044179.1 |
| *P. lemnae* strain L7-75 | 2213 | 2213 | 99 | 94.81 | 1509 | NR_178252.1 |
| *P. wenxiniae* strain 373 | 2204 | 2204 | 99 | 94.73 | 1442 | NR_145946.1 |

**Continued-Table S4.** Partial 16S rRNA sequence alignment results from NCBI BLAST searches for strain YF.

| **Strain** | **Max Score** | **Total Score** | **Query Cover (%)** | **Per. Ident (%)** | **Acc. Len (bp)** | **Accession** |
| --- | --- | --- | --- | --- | --- | --- |
| *P. timonensis* 2301032 | 2206 | 2206 | 99 | 94.73 | 1475 | NR_115198.1 |
| *P. uliginis* N3/975 | 2204 | 2204 | 99 | 94.68 | 1510 | NR_117012.1 |

**Table S5.** Average nucleotide identity (ANI) values and DNA-DNA hybridization (DDH) values compared among YF and 19 related strains.

| **Strain** | **ANI (%)** | **DDH (%)** |
| --- | --- | --- |
| *P. polymyxa* YF | 100 | 100 |
| *P. polymyxa* ATCC15970 | 96.37 | 78.3 |
| *P. polymyxa* CR1 | 96.29 | 76.5 |
| *P. polymyxa* E681 | 96.21 | 74.9 |
| *P. polymyxa* TD94 | 95.96 | 76.9 |
| *P. algicola* HB172198 | 88.85 | 13.4 |
| *P. brasilensis* DSM 14914 | 87.31 | 47 |
| *P. campinasensis* 7537-G1 | 87.09 | 13.4 |
| *P. dakarensis* FF9 | 87.09 | 13.3 |
| *P.farraposensis* pUY79 | 86.74 | 37.6 |
| *P. glucanolyticus* NBRC 15330 | 86.54 | 13.3 |
| *P.hunanensis* DSM 22170 | 84.88 | 13.3 |
| *P. kribbensis* PS04 | 74.88 | 47.6 |
| *P. lemnae* L7-75 | 74.65 | 13.3 |
| *P. macerans* NCTC6355 | 74.64 | 13.3 |
| *P. maysiensis* 1-49 | 74.61 | 46.8 |
| *P. ottowi* MS2379 | 74.53 | 58.7 |
| *P. peoriae* KCTC 3763 | 74.52 | 45.2 |
| *P. terrae* HPL-003 | 74.48 | 46.8 |
| *P. timonensis* DSM 16943 | 74.37 | 13.3 |
|  |  |  |

**Table S6.** Effects of YF on the survival rate of *Codonopsis pilosula*.

| **Index** | **Control group** | | **Treatment group** | | |  |
| --- | --- | --- | --- | --- | --- | --- |
|  | **N-CK** (only *F. oxysporum*) | **P-CK** (*F. oxysporum* + Carbendazim) | **×50** | **×100** | **×150** | |
| Survival rate (%) | 41.72 | 35.10 | 44.24 | 52.07 | 51.31 | |

**Table S7. Expression of disease-causing genes in *F. oxysporum*.**

| **Gene** | **Relative Expression** |
| --- | --- |
| Rho1 | 0.423±0.04 |
| FPD1 | 0.006±0.002 |
| SNF1 | 0.024±0.002 |
| SIX8 | 0.012±0.003 |
| Fmk1 | 0.182±0.04 |
| SIX1 | 0.629±0.03 |
| PL1 | 0.028±0.001 |
| PelD | 0.25±0.02 |

**Table S8.** Statistics of next-generation sequencing data of strain YF.

| **Feature** | **YF** |
| --- | --- |
| Raw pair No. | 3,914,544*2 |
| Raw Base No. (bp) | 587,181,600*2 |
| Raw Q20 (%) | 97.94 |
| Raw Q30 (%) | 94.25 |
| Clean pair No. | 3,888,645*2 |
| Clean Base No. (bp) | 583,296,750*2 |
| Clean Q20 (%) | 98.08 |
| Clean Q30 (%) | 94.42 |
| Coverage (X) | 197 |

**Table S9.** Statistics of third-generation sequencing data of strain YF.

| Items | Value |
| --- | --- |
| Total number of reads | 31,534 |
| Total number of sequenced bases (bp) | 299,807,321 |
| Mean reads length (bp) | 9507.43 |
| Max reads length (bp) | 22,862 |
| N50 (bp) | 10055 |
| Coverage (X) | 51 |

**Table S10.** Statistics of protein coding genes.

| **Feature** | **YF** |
| --- | --- |
| Number of genes | 5138 |
| Total length of genes (bp) | 5,044,320 |
| Average length of genes (bp) | 981.77 |
| Gene density | 0.87 |
| GC content in gene region (%) | 46.73 |
| gene/genome (%) | 85.45 |
| Total length of intergenetic region (bp) | 859036 |

**Table S11.** Statistics of gene islands (GIs).

| **Island ID** | **Island Start** | **Island End** | **Length (bp)** | **CDS No.** |
| --- | --- | --- | --- | --- |
| GI01 | 1,144,279 | 1,163,831 | 19,552 | 13 |
| GI02 | 1,310,856 | 1,325,976 | 15,120 | 16 |
| GI03 | 1,647,200 | 1,659,678 | 12,478 | 7 |
| GI04 | 2,601,416 | 2,681,779 | 80,363 | 43 |
| GI05 | 2,767,582 | 2,794,508 | 26,926 | 24 |
| GI06 | 3,016,363 | 3,037,566 | 21,203 | 25 |
| GI07 | 3,540,240 | 3,556,156 | 15,916 | 19 |
| GI08 | 3,830,419 | 3,859,163 | 28,744 | 42 |
| GI09 | 539,631 | 544,981 | 5,350 | 10 |
| GI10 | 5,583,586 | 5,604,391 | 20,805 | 31 |

**Table S12.** Statistics of Clustered Regularly Interspersed Short Palindromic Repeats (CRISPRs).

| **ID** | **Start (bp)** | **End (bp)** | **Length (bp)** | **DR No.** | **DR Average Len (bp)** | **SPA Average Len (bp)** |
| --- | --- | --- | --- | --- | --- | --- |
| CRISPR1 | 1,166,105 | 1,166,510 | 406 | 6 | 46 | 26 |
| CRISPR2 | 2,274,237 | 2,275,736 | 1,500 | 23 | 32 | 34 |
| CRISPR3 | 2,276,074 | 2,276,235 | 162 | 3 | 30 | 36 |
| CRISPR4 | 2,285,333 | 2,286,183 | 851 | 13 | 33 | 35 |
| CRISPR5 | 2,292,669 | 2,293,502 | 834 | 13 | 32 | 34 |
| CRISPR6 | 2,463,179 | 2,465,823 | 2,645 | 28 | 26 | 71 |
| CRISPR7 | 4,376,576 | 4,376,887 | 312 | 4 | 33 | 60 |
| CRISPR8 | 5,138,701 | 5,138,878 | 178 | 3 | 28 | 47 |

**Table S13.** Putative gene clusters that encoded for secondary metabolites in YF.

| **Gene clusters** | **Types** | **Most Similar Known Clusters** | **Location** | | **Size (bp)** | **Similarity to Known Clusters (%)** | **Similarity to clusters identified in related species (%)** | **Bioactivity** |
| --- | --- | --- | --- | --- | --- | --- | --- | --- |
|  |  |  | **Start** | **End** |  |  |  |  |
| Cluster 1 | Polyketide, NRPS | BGC0001089 | 67,496 | 131,241 | 63,746 | 100 | 97 | Fusaricidin B |
| Cluster 2 | Siderophore | - | 1,090,990 | 1,126,394 | 35,405 |  | 100 | Ni-Siderophore |
| Cluster 3 |  | - | 1,257,583 | 1,277,819 | 20,237 |  | 85 | Proteusin |
| Cluster 4 | RiPP | BGC0001356 | 1,383,363 | 1,407,485 | 24,123 | 40 | 100 | Paeninodin |
| Cluster 5 | NRPS | BGC0001469 | 1,462,635 | 1,523,724 | 61,090 | 41 | 95 | Marthiapeptide |
| Cluster 6 | RiPP | BGC0001727 | 1,715,676 | 1,742,683 | 27,008 | 100 | 100 | Paenilan |
| Cluster 7 | NRPS-like | - | 2,045,352 | 2,089,047 | 43,696 |  | 100 | - |
| Cluster 8 | RiPP | - | 2,095,192 | 2,115,835 | 20,644 |  | 94 | Cyclic-lactone-autoinducer |
| Cluster 9 | NRPS | BGC0000449 | 2,485,992 | 2,568,243 | 82,252 | 80 | 59 | Tridecaptin |
| Cluster 10 | NRPS | BGC0000400 | 2,621,277 | 2,697,416 | 76,140 | 60 | 59 | Paenibacterin |
| Cluster 11 | NRPS | - | 2,834,179 | 2,886,938 | 52,760 |  | 85 | Betalactone |
| Cluster 12 | RiPP | - | 2,934,627 | 2,955,175 | 20,549 |  | 100 | Cyclic-lactone-autoinducer |
| Cluster 13 | Polyketide, NRPS | BGC0001089 | 3,448,222 | 3,549,953 | 101,732 | 57 | 65 | Bacillaene |
| Cluster 14 | NRPS | BGC0000408 | 4,761,617 | 4,842,662 | 81,046 | 100 | 80 | Polymyxin B |
